# Interpretable prediction of necrotizing enterocolitis from machine learning analysis of premature infant stool microbiota

**DOI:** 10.1186/s12859-022-04618-w

**Published:** 2022-03-25

**Authors:** Yun Chao Lin, Ansaf Salleb-Aouissi, Thomas A. Hooven

**Affiliations:** 1 Department of Computer Science, Columbia University, 1214 Amsterdam Ave., Mailcode 0401, New York, 10027 USA; 2 Department of Pediatrics, University of Pittsburgh School of Medicine, Pittsburgh, USA; 3 Richard King Mellon Institute for Pediatric Research, UPMC Children’s Hospital of Pittsburgh, Pittsburgh, USA

**Keywords:** Necrotizing enterocolitis, Multiple instance learning, Prematurity, Microbiome

## Abstract

**Background:**

Necrotizing enterocolitis (NEC) is a common, potentially catastrophic intestinal disease among very low birthweight premature infants. Affecting up to 15% of neonates born weighing less than 1500 g, NEC causes sudden-onset, progressive intestinal inflammation and necrosis, which can lead to significant bowel loss, multi-organ injury, or death. No unifying cause of NEC has been identified, nor is there any reliable biomarker that indicates an individual patient’s risk of the disease. Without a way to predict NEC in advance, the current medical strategy involves close clinical monitoring in an effort to treat babies with NEC as quickly as possible before irrecoverable intestinal damage occurs. In this report, we describe a novel machine learning application for generating dynamic, individualized NEC risk scores based on intestinal microbiota data, which can be determined from sequencing bacterial DNA from otherwise discarded infant stool. A central insight that differentiates our work from past efforts was the recognition that disease prediction from stool microbiota represents a specific subtype of machine learning problem known as multiple instance learning (MIL).

**Results:**

We used a neural network-based MIL architecture, which we tested on independent datasets from two cohorts encompassing 3595 stool samples from 261 at-risk infants. Our report also introduces a new concept called the “growing bag” analysis, which applies MIL over time, allowing incorporation of past data into each new risk calculation. This approach allowed early, accurate NEC prediction, with a mean sensitivity of 86% and specificity of 90%. True-positive NEC predictions occurred an average of 8 days before disease onset. We also demonstrate that an attention-gated mechanism incorporated into our MIL algorithm permits interpretation of NEC risk, identifying several bacterial taxa that past work has associated with NEC, and potentially pointing the way toward new hypotheses about NEC pathogenesis. Our system is flexible, accepting microbiota data generated from targeted 16S or “shotgun” whole-genome DNA sequencing. It performs well in the setting of common, potentially confounding preterm neonatal clinical events such as perinatal cardiopulmonary depression, antibiotic administration, feeding disruptions, or transitions between breast feeding and formula.

**Conclusions:**

We have developed and validated a robust MIL-based system for NEC prediction from harmlessly collected premature infant stool. While this system was developed for NEC prediction, our MIL approach may also be applicable to other diseases characterized by changes in the human microbiota.

**Supplementary Information:**

The online version contains supplementary material available at 10.1186/s12859-022-04618-w.

## Background

Premature infants are a high-risk medical population, subject to numerous complications of physiologic underdevelopment. Among premature infants, one of the most serious illnesses is necrotizing enterocolitis (NEC). Patients with NEC develop progressive inflammation and necrosis of the intestinal wall, which can quickly spread from an initial focus of injury to involve the entire intestine.

NEC is a common emergency in the neonatal intensive care unit (NICU), affecting 5–15% of very low birth weight (<1500 g; VLBW) infants [1, 2], with peak incidence around 30 weeks corrected gestational age [3, 4]. Mortality rates from NEC are 18–53% [5–9]. Survivors are at risk for multiple serious, long-term complications such as nutritional deficiencies, recurrent infections, liver failure, and cognitive and motor developmental impairments [7, 10, 11].

There is no widely available, reliable biomarker or other clinical test for determining an individual patient’s risk of NEC. The current standard-of-care is to closely observe all preterm infants for clinical signs of NEC, which may include abdominal swelling, vomiting, bloody stools, and vital sign instability [12, 13]. Unfortunately, even with close clinical monitoring, some infants are diagnosed only after the disease has progressed significantly.

**Table 1:** Shared clinical features from the two studies included in the MIL model

	Warner et al. (n = 161)			Olm et al. (n = 100)		
	Non-affected (116)	NEC affected (45)	*P*-value$^{\mathrm{a}}$	Non-affected (70)	NEC affected (30)	*P*-value$^{\mathrm{a}}$
Mean gestational age at birth (SD)	27.1 (2.3)	26.3 (2.5)	0.10	28.1 (2.3)	28.2 (2.3)	0.83
Percent male	0.45	0.62	0.05	0.47	0.4	0.66
Mean birthweight (SD)	963.7 (268.7)	856.8 (243.1)	**0.03**	1092.6 (343.2)	1076.1 (341.6)	0.83
Percent vaginal	0.31	0.27	0.70	0.26	0.30	0.81
Percent multiple gestation	0.28	0.18	0.23	0.33	0.53	0.07
Number live-born$^{\mathrm{b}}$	1–3	1–3	N/A	1–3	1–3	N/A
Percent exclusively formula fed	0.03	0.02	> 0.99	0.11	0.10	> 0.99
Percent exclusively breast fed	0.21	0.33	0.10	0.30	0.37	0.64
Percent combination breast and formula fed	0.77	0.62	0.08	0.59	0.53	0.66
Percent NPO$^{\mathrm{c}}$ throughout hospitalization	0.00	0.02	0.28	0.00	0.00	> 0.99

*P*-value < 0.05 is in bold

$^{\mathrm{a}}$Student’s *t*-test for mean value comparisons; Fisher’s exact test for percentage comparisons

$^{\mathrm{b}}$Singleton infants = 1; for multiple gestations, the number designates live-born birth order

$^{\mathrm{c}}$NPO = nothing *per os* (not being fed)

A preferable strategy would be to develop and apply a form of predictive monitoring to assign dynamic risk scores that could be followed over time. Such predictive monitoring could facilitate early identification of patients whose risk was increasing without waiting for the disease to occur. Prompt, early initiation of therapy in response to a reliable indicator of rising risk could reduce rates of serious NEC complications, including death [2, 14–17].

The causes of NEC are multifactorial and not completely understood. One factor implicated in NEC pathophysiology is the intestinal microbiota. The intestinal lumen is sterile or nearly sterile at birth, but becomes colonized by bacteria during the first days of postnatal life [18–20]. Several groups have shown that there is a stereotyped succession of colonizing taxa in healthy infants [19, 21]. Intestinal colonization of preterm newborns is more complex, less predictable, and more easily disrupted due to various factors, however. Prolonged hospitalization, invasive instrumentation, and recurrent exposure to antenatal and postnatal antibiotics alter the trajectory of the preterm intestinal microbiota [22–24].

Culture-free, sequence-based characterization of stool bacteria can serve as a noninvasive proxy for classifying the intestinal microbiota [25, 26], and several previous studies have examined neonatal stool for patterns of bacterial colonization associated with NEC. These investigations have revealed correlations between microbiota characteristics and development of NEC, although the details have not been fully congruent. Several authors have identified a relative abundance of bacteria in the family Enterobacteriaceae preceding NEC [27–29], which may in turn reflect the abundance of Enterobacteriaceae-binding antibodies in breastmilk [30]. Others have shown that NEC correlates with below-average abundance of bacteria in the class Negativicutes [31] or phylum Firmicutes [29]. More specific bacterial taxa, such as members of the genus *Klebsiella* have also been implicated as contributors to NEC pathophysiology [27, 28]. These correlations, while informative to a fuller understanding of NEC risk factors, have not proven strong enough to establish reliable test characteristics that could be used to predict NEC in an individual patient.

We hypothesized that machine learning (ML) techniques applied to neonatal intestinal microbiome data from otherwise discarded stool samples could generate a clinically useful NEC risk score. We suspected that early changes in the microbiota might be detectable to certain ML algorithms, permitting delineation of high risk populations well before disease onset.

Other teams have used ML approaches to try to determine NEC risk factors. One group used a linear discriminant analysis on clinical, laboratory, and radiographic signs of NEC in order to develop a prognostic algorithm to predict disease outcomes [32]. Olm et al. applied a boosted gradient classifier to preterm microbiota data to identify bacterial taxa that precede NEC [27]. While these efforts have been productive, no published report has systematically evaluated ML strategies for converting microbiota data into an actionable NEC risk score. This may be because of challenges inherent in microbiota data derived from next-generation sequencing. Such datasets are intrinsically sparse, non-normal, compositional, and have high dimensionality [33–36], all of which can obscure subtle signals and lead to suboptimal ML outcomes.

In this report, we detail development, testing, and application of a system for sequential quantification of a preterm infant’s risk of NEC using information about the bacterial content of the stool. We developed and validated our approach using historical datasets from two large cohort-based studies of preterm infant stool microbiota. This work builds on a preliminary description of our efforts, which we reported in 2020 [37]. Significant advances reported here include refinement of our data pre-processing approach, development of a quantitative risk score, further investigation of interpreting the major drivers of NEC prediction, and addition of a second, independent patient database.

Our system is organized around a gated attention-based multiple instance learning (MIL) model. MIL is a framework for approaching problems where the goal is to learn to label sets of instances even when the individual instances themselves are not inherently labeled [38]. We propose that MIL offers strategies well-suited for predicting a disease based on serial microbiota characteristics. To our knowledge, no prior research has investigated this strategy, which we show permits accurate risk discrimination from the high-dimensionality, low signal strength data generated by stool microbiota classification.

We also introduce a novel technique that we term the “growing bag” analysis to apply MIL to a longitudinal set of clinical samples. The growing bag analysis allows us to convert confidence scores from our MIL model into a dynamic risk score that quantifies an individual patient’s likelihood of developing NEC.

Finally, to interpret dynamic changes to NEC risk revealed through the growing bag analysis, we employ the gated attention mechanism within our system, which we use to identify individual stool samples from patients in our two cohorts that are maximally informative to NEC risk. We apply random forest analysis to these high-attention samples to identify a core set of colonizing bacterial taxa that inform our system’s risk assessment. This set of bacteria—which include taxa previously implicated by other researchers as well as novel taxa not previously reported to be associated with NEC risk—could be the basis for a future, ML-based bedside assay for identifying infants at elevated NEC risk in real time.

## Results

### Microbiome characterization of longitudinal preterm infant stool samples from two independent, historical NICU cohorts

We developed and validated our ML NEC risk assessment system using data from two large studies of preterm infants from which stool microbiota DNA sequences and basic clinical details—including NEC outcomes—were available [27, 31].

**Fig. 1:**
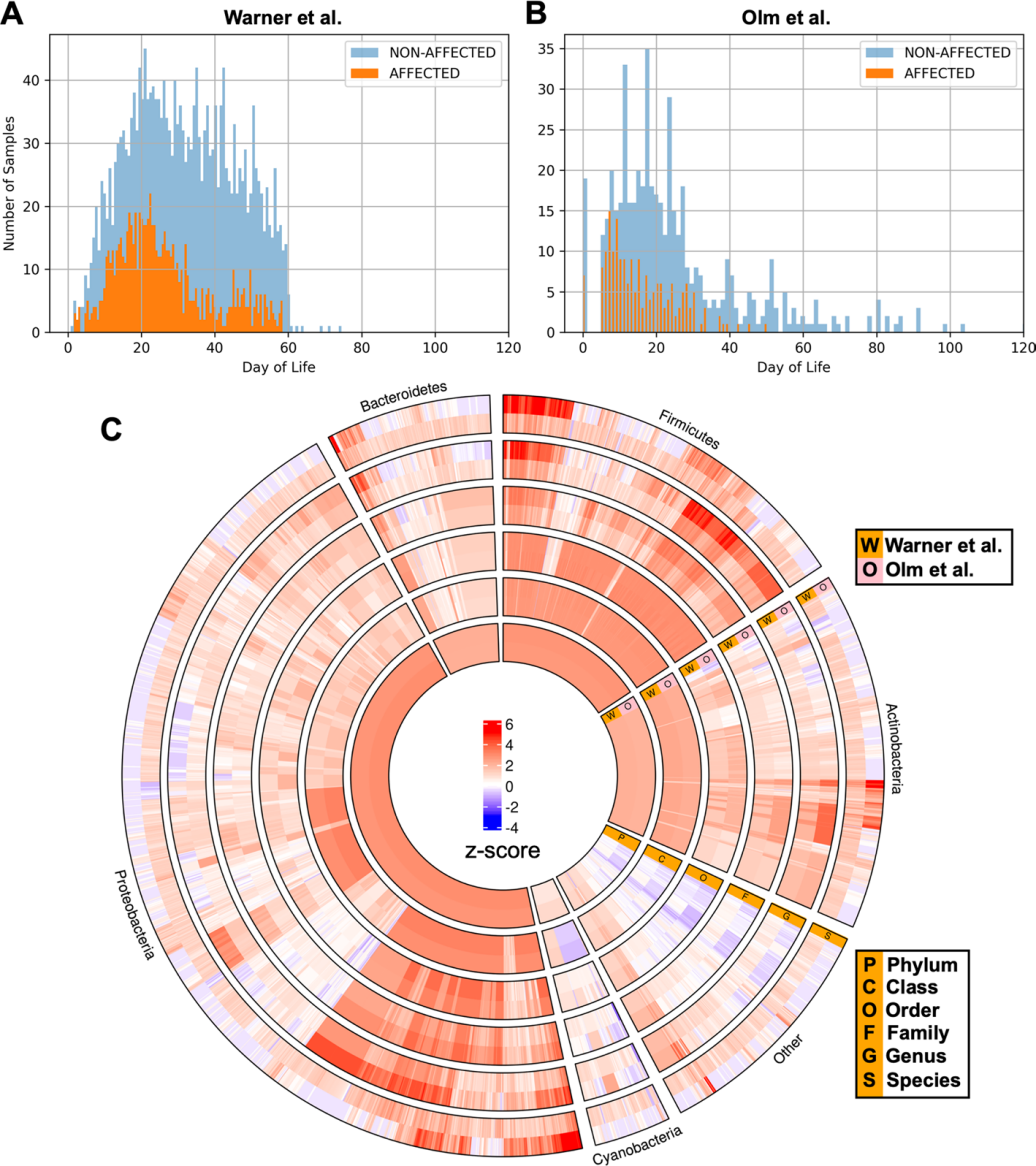
Collection timing and microbiota characterization of neonatal stool samples from the two cohorts used for NEC prediction. Warner et al. [31] and Olm et al. [27] prospectively collected stool samples for subsequent sequence-based microbiota analysis from hospitalized preterm infants at variable timepoints throughout the first several months of life. A subset of enrolled infants eventually developed NEC. For this study, 2895 samples from Warner et al. and 700 from Olm et al. were used for development and retrospective application of a NEC prediction model (**A**, **B**). Kraken2 was used to perform microbiota member classifications for all available samples from both studies. Aligned bacterial classifications at all taxonomic levels are shown for both studies (**C**)

The designs of the two studies were conceptually similar. Warner et al. [31] prospectively collected serial stool samples and recorded a panel of clinical data from VLBW preterm infants cared for at three U.S. hospitals. NEC cases were identified based on clinical and radiographic features, and were matched to between one and four control infants who did not develop NEC. Case-control matching was based on gestational age at delivery and the birth medical center. All stools from birth through disease onset were analyzed for NEC-affected cases, whereas stools between birth and 74 days of life were analyzed for controls (the majority of control patients had samples collected up to day of life 60, see Fig. 1A). To characterize the microbiota in collected stools, Warner et al. used Roche 545 sequencing of 16S ribosomal RNA variable region amplicons.

Olm et al. [27] also prospectively collected preterm infant stool and recorded clinical data over the first months of life, then performed post-hoc case-control matching based on gestational age at delivery and the calendar date at birth (to control for potential seasonal fluctuation). Infants for this study were all cared for in the NICU at University of Pittsburgh Medical Center Magee-Womens Hospital. There was no overlap between patient populations for the two studies. As in the Warner et. al. study, NEC-affected infant stool was analyzed between birth and disease onset, with samples preferentially selected for sequencing if they were the last collected before NEC diagnosis (Fig. 1B). In this study, control stool samples selected for analysis were matched in time to case samples. Instead of targeted sequencing of the 16S ribosomal RNA coding region, Olm et al. performed whole-genome sequencing on an Illumina HiSeq 2500 platform, permitting reconstruction of complete or near-complete bacterial genomes. Whole-genome sequencing allows greater precision in bacterial taxonomic classification and reconstruction of metabolic pathways present among the bacterial population than targeted 16S sequencing, but requires more sequencing and bioinformatics resources [39, 40].

We obtained authorized access to Warner et al. patient metadata through the National Center for Biotechnology Information’s database of genotypes and phenotypes (NCBI dbGAP accession phs000247.v5.p3) and used metadata published by Olm et al. Patient details, which included NEC outcomes, were matched with publicly available next-generation sequencing files, which we downloaded from the NCBI’s Sequence Read Archive. Because the two studies used different sample collection protocols, next-generation sequencing strategies, and reported varying demographic and clinical information, we took several steps to ensure that data we used from the two sources were normalized and structured identically.

FASTQ files with 16S ribosomal RNA variable region sequences (Warner et al. dataset) and whole genome shotgun sequences (Olm et al. dataset) were characterized with Kraken2, a flexible microbiota classification software package that aligns short genetic sequences (k-mers) to the lowest common ancestor in order to make a taxonomic assignment [41]. We chose Kraken2 for its speed and resource-efficiency and the fact that it produces identically formatted microbiota classification files regardless of whether its input consists of 16S or whole genome sequences. This uniformity is well-suited for downstream ML analyses, permitting a single model to accept data from distinct study protocols.

After removing all non-bacterial taxa (fungi and archaea) from the two collections of Kraken2 output files, we were left with bacterial DNA fragment counts for stool microbiota from 3595 samples taken from 261 preterm infants (2895 samples from 161 infants from the Warner study, 700 samples from 100 infants from Olm et al.), 75 of whom developed NEC. The taxonomic distribution of the bacteria identified through this analysis is presented in Fig. 1C. Trends identified in both source studies reemerged after our reclassification of the stool microbiota using Kraken2. Specifically, we found that infants from the Warner et al. cohort who developed NEC had a trend toward decreased Negativicutes abundance and increased Proteobacteria abundance relative to unaffected controls. We also found an increase in *Klebsiella pneumoniae* strain 242_2 among the Olm et al. NEC cases preceding disease onset, which those authors described in their original report (Additional file 1: Fig. S1).

Both Warner et al. and Olm et al. reported demographic and clinical information about enrolled neonates and their families. These metadata ranged from maternal information such as age, parity, and biometric data, to details about the birth—such as mode of delivery—to neonatal metadata such as birthweight, age at the time of each sample collection, and Apgar scores. The collected metadata categories differed between the two studies, however. For our ML analysis, we elected to use a limited metadata collection representing the intersection of the two studies (Table 1).

### Pre-processing of compositional microbiome feature counts normalizes data and prunes uninformative taxa

Microbiota classification data generated through next-generation sequencing have characteristics that present challenges to ML-based analyses. First, stool microbiota datasets are sparse: the majority of potential bacteria will not be found in any given sample, resulting in large numbers of “zero count” organisms [36, 42]. Furthermore, microbiota classification data have a characteristic known as compositionality. Briefly, because the total number of DNA reads that can be generated by a next-generation sequencing instrument is finite, any next-generation sequencing-based taxonomic classification is necessarily based on a subsample of the total environmental diversity. As a result, a microbiota community member characterized as absent (i.e. zero on the count table) may actually be present at a low density below the instrument’s limit of detection. Furthermore, incorrect assumptions about correlations between microbiota member abundances can result if the compositional nature of the data is not considered [34–36, 43, 44].

**Fig. 2:**
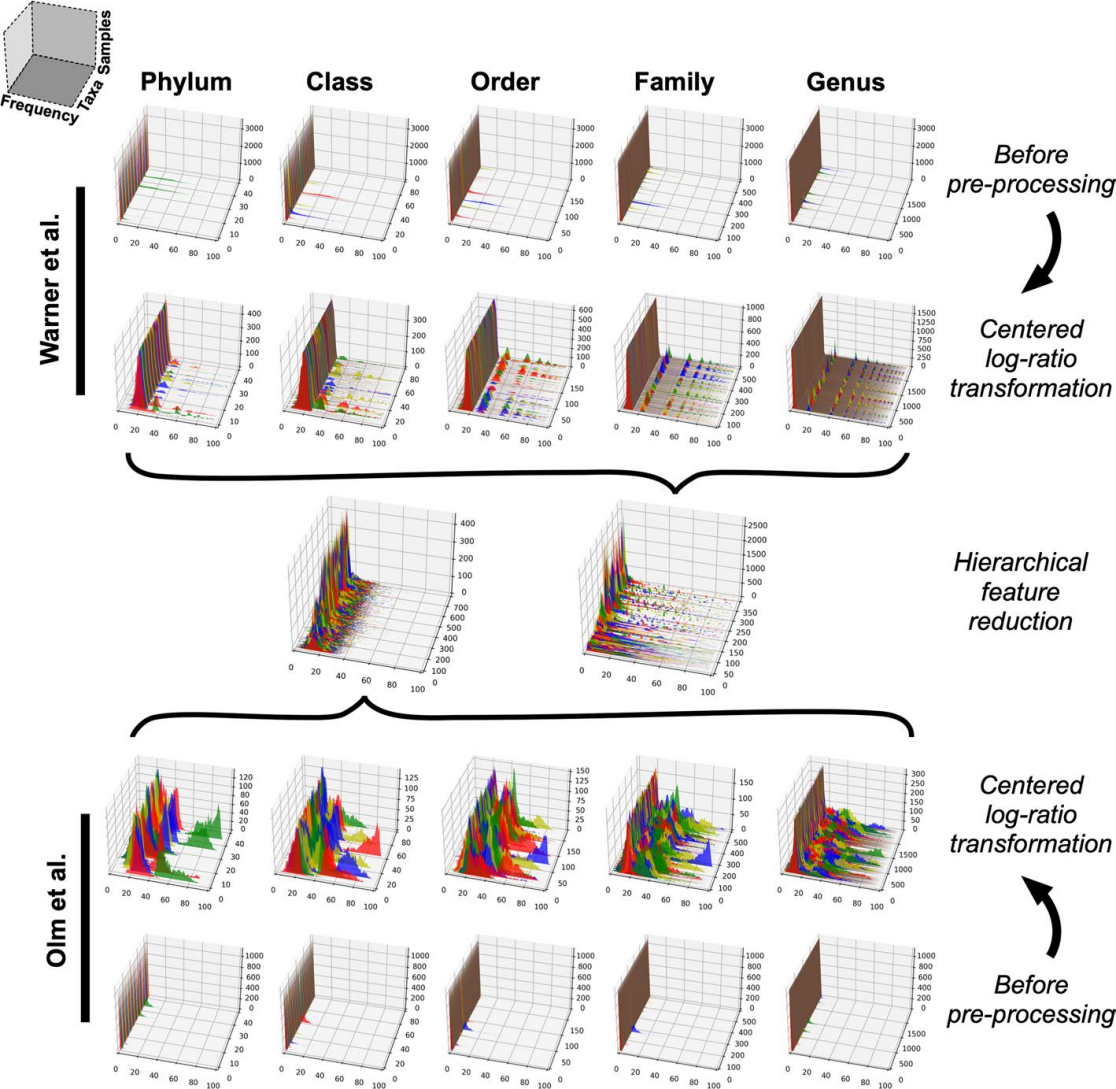
Microbiota data pre-processing. Kraken2 bacterial taxa raw counts were processed in two steps before use as input for MIL NEC prediction. Centered log-ratio transformation (step 1) replaces absent taxa with a non-zero value (0.66) and accentuates differences between sparse data collections. This was followed by hierarchical feature reduction to reduce data dimensionality by algorithmically removing uninformative bacterial taxa (step 2). Hierarchical feature reduction involved pruning all branches of the taxonomic tree whose abundance showed $>0.7$ Pearson correlation with their parent nodes or which yielded no information gain toward NEC classification. The same processes were applied to both the Warner et al. and the Olm et al. microbiota datasets. For each plot in the figure, the X axis describes a normalized frequency distribution while the Y axis describes the number of patient samples. The Z axis describes the bacterial taxa present at each stage of pre-processing (the total number decreases after step 2; see main text). Peaks are colored with a repeating, alternating pattern for ease of visualization. More abundant taxa are those with higher peaks toward the right side of the plot

A common approach for handling the uncertain zeros in a compositional dataset is to apply a pseudocount where zeros are replaced by a uniform small number, such as two-thirds of the limit of detection [45, 46]. For our dataset, zeros were replaced with 0.66, which was two-thirds of the smallest possible read count of 1. Next, we applied a centered log-ratio transformation in order to adjust compositional data to make them more tractable to statistical analyses. In the centered log-ratio approach, each taxon within a sample is transformed by taking the log-ratio counts for that taxon within a sample divided by the geometric mean of the counts of all taxa [47, 48]. This transformation adjusts sparse datasets so that the frequency distributions are not tightly clustered close to zero; it shifts and widens narrow frequency distributions, accentuating subtle differences that may be lost in the non-transformed data (see Fig. 2).

Another special characteristic of microbiota data is its hierarchically structured feature space (bacteria can be classified at kingdom, phylum, class, order, family, genus, and species levels), which can be exploited for more efficient and accurate modeling through taxonomy dimensionality reduction [49, 50]. To reduce the dimensionality of our taxonomic microbiota data, we removed any node that was redundant with its parent, using a Pearson correlation threshold of 0.7. This reduced the the number of taxa from 3702 to 2282 for the Warner et al. dataset and from 9626 to 6221 for the Olm et al. dataset. Information gains were also calculated for each node of the taxonomy tree using the NEC target label. Any node with an information gain of zero was discarded. This process allowed us to prune the number of features further to 362 taxa and 706 taxa for the Warner and Olm datasets, respectively. For a list of taxa included in the final analyses, after pre-processing, see Additional file 2: Data S1.

Pre-processing highlighted inherent differences between the two datasets. The Warner et al. study, which used relatively low-resolution 16S sequencing, generated a sparser, lower-complexity data landscape after centered log-ratio transformation than the Olm et al. study, in which whole genome sequencing generated greater phylogenetic differentiation and more nuances in the data landscape (Fig. 2). However, the hierarchical feature reduction step removed low-information taxa from both datasets, resulting in similar feature frequency landscapes from the two studies. A significant strength of the NEC prediction system we describe here is that it performed well with different degrees of data complexity, suggesting that it can be successfully paired with various technical strategies for microbiota characterization.

### An attention-based multiple instance learning paradigm permits discrimination between affected and unaffected infants using pre-processed microbiome data and basic sample metadata

The field of ML offers a wide range of algorithms to build models from data. In most cases, ML requires training from a set of discrete data points, which are termed instances. Each instance is described by a set of feature values in some domain, such as text for emails, pixel intensity for images, or vital signs for patients. Often, each instance is tagged with its own label (for example, spam or non-spam email). For any new ML task, a crucial step is to identify the learning paradigm and the representation needed to formalize the problem. This will depend on the data and the goal.

MIL is a form of ML where the training instances are arranged in sets, called bags, and the label is provided for the entire bag [51, 52]. There are two main approaches to solve MIL problems. In *instance level approaches*, predictions are made for each instance and aggregated to obtain the bag level label. In *embedding level approaches*, instances are mapped to a vectorial embedded space and fed to a final classifier. Embedding level approaches are preferred when instance-level labels are unknown, which is the case for NEC prediction, where no single stool sample collected prior to disease onset can be definitively classified as NEC-affected or unaffected.

**Fig. 3:**
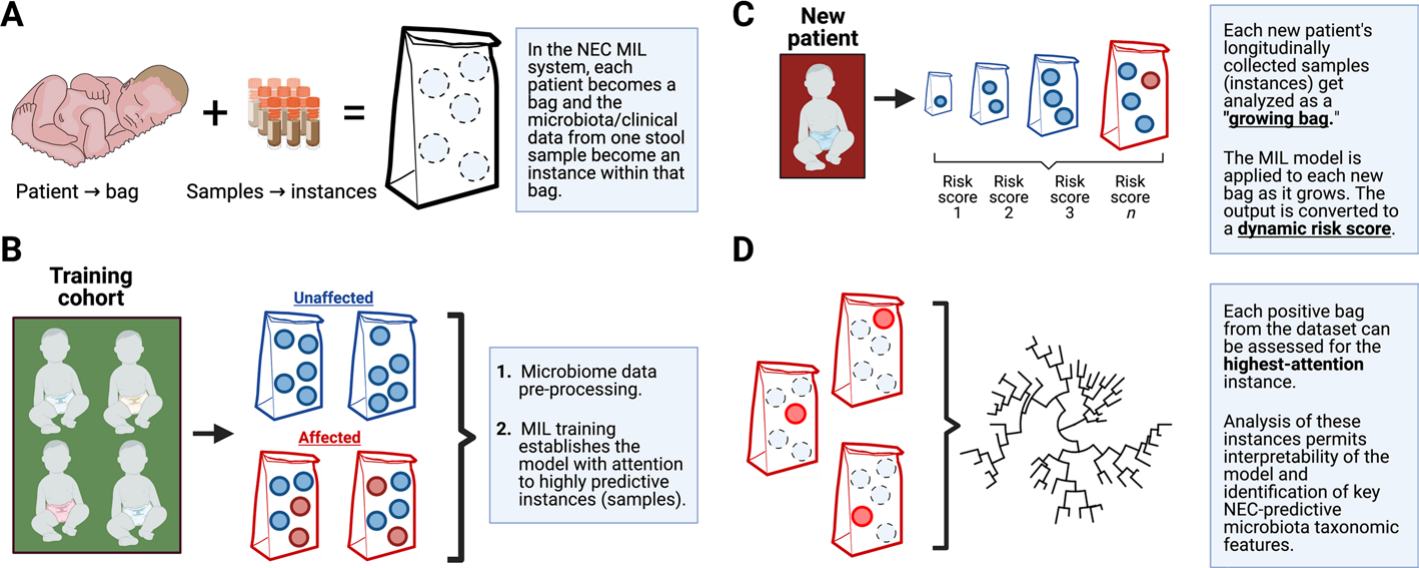
Multiple instance learning (MIL) approaches employed in this study. In bag-level MIL, each patient is represented as a bag of multi-feature instances, which are stool samples (the instances) that have been characterized by microbiota states and accompanying basic clinical data (the features). The instances are inherently unlabeled and the goal of the system is to determine a bag label (**A**). The NEC prediction model was trained on pre-processed microbiota frequency data and basic clinical data from training patient cohorts. This permitted development of a model for bag classification and quantifiable attention to key instances with highest contributions to bag labels (**B**). Test patients were assessed with the trained model, which was naïve to their data, using a growing bag approach where each new instance generated a new confidence score and attention distribution across all available instances. The changing confidence scores were algorithmically transformed into dynamic risk scores for each test patient (**C**). Attention scores were also used to identify key, highly informative instances. The feature distributions within these instances were subjected to random forest analysis to identify specific bacterial taxa that drove accurate NEC prediction (**D**)

We recognized MIL as an appropriate framework for NEC prediction from serial stool sample microbiota analysis. In adapting a MIL approach to predicting NEC, we model each patient as a bag of clinical samples, which are the instances. The instances have feature values that consist of microbiota data and associated clinical details (Table 1). The task is to assign a NEC risk classification to the patient (Fig. 3A) based on the unlabeled instances.

We used a recently developed embedding level strategy in which initial embedding of the features is performed by a neural network, which feeds into an attention-gated MIL pooling algorithm [53]. The embeddings are aggregated using the attention weights, which are then passed to a fully connected layer with a sigmoid activation function that produces a bag probability. This model assumes that a bag label $X$ is distributed as a Bernoulli distribution $\theta (X) \in [0, 1]$ and trains it by optimizing the log likelihood function (see Additional file 3: Fig. S2).

The attention-based pooling module of our MIL system assigns attention weights to each instance in a bag. Those instances with the highest attention weights are the most significant contributors to the final bag label. This allows identification of maximally predictive stool samples (Fig. 3B). We used attention weight data to tune dynamic NEC risk scores and to interpret the microbiota taxonomic features that were most informative to our model.

We used stratified sampling to partition our two datasets into training and testing sets. We conducted five trials of this partitioning to avoid any sampling bias. We used a cross-validation modeling strategy whereby we trained our system on a portion of the dataset, then repeatedly tested the resultant model on the data subset that was withheld during training. The final model obtained was then applied to the test set. We averaged the results across all trials. We compared our system to two instance-level MIL approaches called *mi-SVM* and *MI-SVM* [54]. We also compared to *MILboost*, a MIL variant of AdaBoost that has been mostly used for object detection in images [55]. Finally, we compared to logistic regression. The PyTorch Python package [56] was used to implement the attention-based MIL method. The Scikit-learn Python package was used to implement SVM and logistic regression analyses [57].

**Fig. 4:**
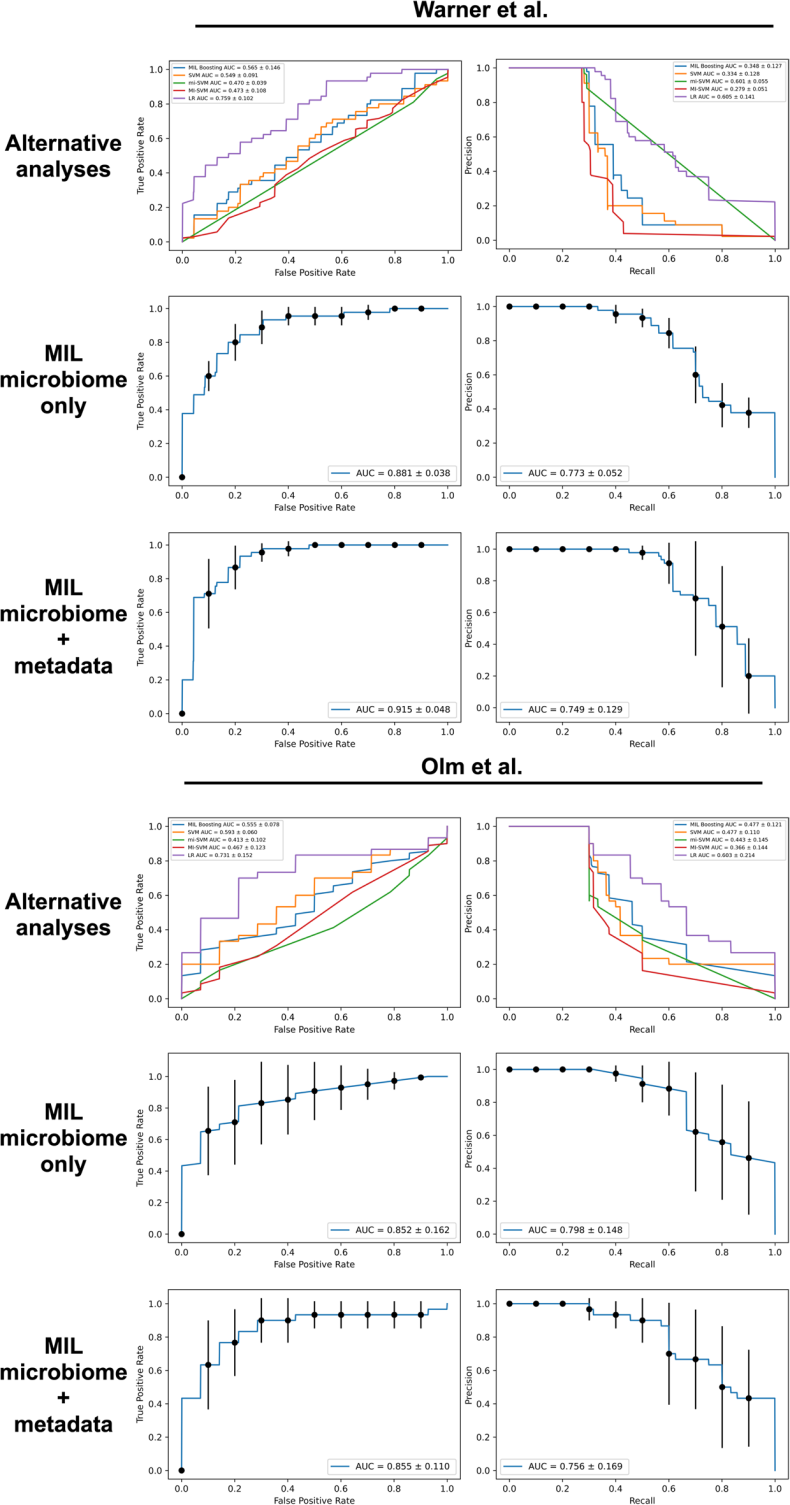
Receiver-operator and precision-recall curves for comparison methods and the multiple instance learning (MIL) model with and without clinical metadata. Curves are displayed for both clinical datasets using five comparison techniques (top panels) for which the average curves from five testing trials are displayed. The MIL microbiome and microbiome-plus-metadata panels show average curves from five testing trials with 95% confidence intervals in parentheses. AUC, area under curve; SVM, support vector machine; LR, logistic regression

In repeated trials of our attention-based MIL approach, we demonstrated receiver-operator curve (ROC) areas under the curve (AUC) of 0.86–0.92 for both datasets, suggesting a good balance of sensitivity and specificity. Precision-recall characteristics of the experimental system also exceeded any of the alternative methods, with precision-recall AUC values around 0.75 (Fig. 4). The majority of our model’s predictive performance came from microbiome data. Exclusion of clinical and demographic details in Table 1 from the analysis resulted in AUC values that were not significantly different than when these metadata were included ($p\ge 0.35$ for Kolmogorov–Smirnov comparisons between ROC and precision-recall curves with and without clinical data). Pre-processing as described above was essential, however. Dimensionality reduction through hierarchical feature and information gain-based pruning allowed our models to converge, which was not possible when trained with the complete microbial dataset. Centered log-ratio transformation increased the ROC and precision-recall AUCs (Additional file 4: Fig. S3). By contrast, the predictive accuracy of the alternative approaches was barely above chance, with receiver-operator characteristics demonstrating only limited ability to differentiate NEC from non-NEC cases (Fig. 4).

### A novel “growing bag” method permits determination of a NEC risk score from serial analysis of neonatal stool samples

When supplied with historical data spanning early infancy, our MIL-based system effectively distinguishes unaffected preterm newborns from those who developed NEC. However, a more useful clinical tool would be able to make dynamic predictions over time.

In order to generate dynamic NEC predictions using our system, we developed an approach that we call the growing bag analysis. Here, the model is initially trained on the full set of complete bags except for the bag of interest, representing the patient whose longitudinal NEC prediction is under study. That patient’s instances are then arranged in sequential order and the trained model is asked to generate iterative NEC predictions where each new prediction represents the previous bag to which one new instance has been added (i.e. the growing bag). The process can be repeated for every patient to produce NEC confidence scores as a function of time (Fig. 3C).

**Fig. 5:**
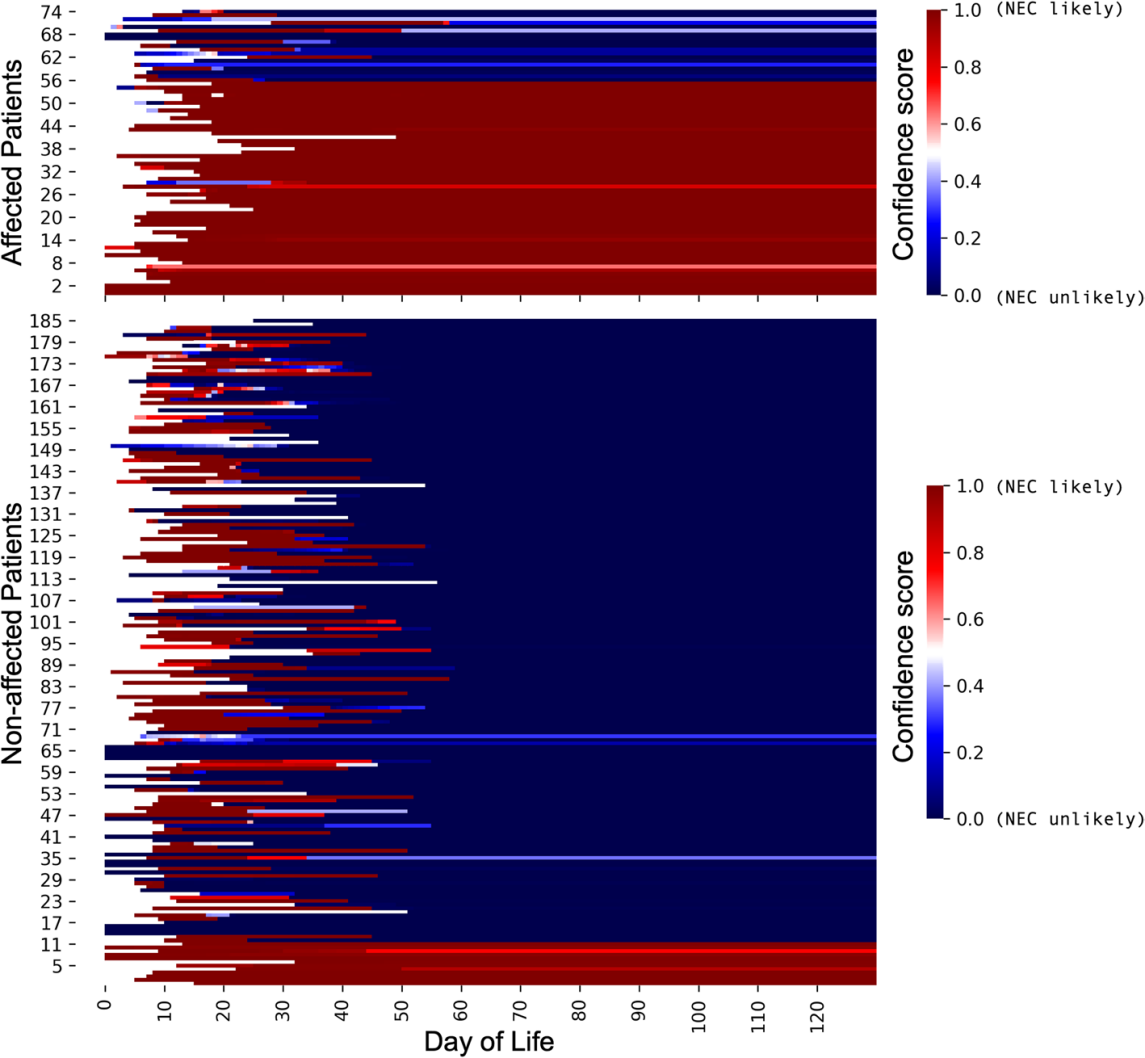
Confidence scores for all patients using the growing bag MIL technique. Confidences associated with MIL NEC prediction were calculated for all available samples using the growing bag technique as described in the main text. In this figure, a prediction of NEC where the confidence is high is colored red, while a prediction with low confidence is colored blue. Gaps between samples taken from the same patient were interpolated with the confidence of the previously obtained sample. The order of patients along the left side of this figure is the same as in Fig. 6D

Plotting the confidence-over-time functions for patients in our historical datasets revealed an unexpected pattern. The system indicated that the majority of preterm newborns were predicted to be NEC-positive at birth. Over time, however, the two groups diverged, with NEC-negative patients showing a sudden drop in the system’s confidence that they would develop the disease. Meanwhile, most of the affected patients did not show this sudden inflection; their prediction remained NEC-positive throughout the observation period (Fig. 5).

**Fig. 6:**
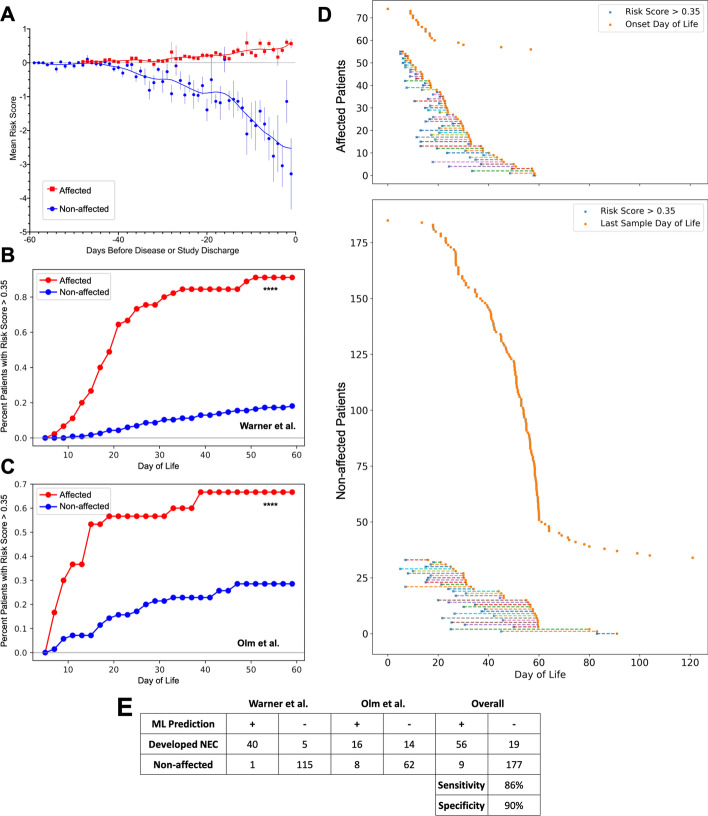
NEC risk scores and test characteristics. Confidence scores from the growing bag were used to calculate dynamic NEC risk scores as described in the main text. Each patient in both clinical cohorts was analyzed individually, using a MIL model that was naïve to that patient’s data. Aggregate risk scores for all patients were binned by days preceding disease or study discharge, and are displayed with standard deviation. The trend lines were generated by LOWESS curve fitting (**A**). Separate survival curves were generated for patients in the Warner and Olm cohorts (**B**, **C**) where the outcome of interest was a risk score above 0.35 during hospitalization. Differences between affected and non-affected patient curves were statistically significant (****$p<0.001$; Kolmogorov–Smirnov test) for both cohorts.Timelines for all patients from both cohorts are labeled with the last sample collected (**D**). For those who were predicted to have NEC based on a risk score >0.35, the first sample to cross the risk threshold is indicated, with dotted lines illustrating lead-time before disease onset (for true positives) or the last sample collected before aging out of the study (for false positives). Prediction outcomes for all patients used across both cohorts are displayed in the confusion matrix (**E**)

We next developed a transformation that converts the MIL model’s confidence score into a risk score, which is also influenced by the attention weight assigned to each new instance. The algorithm incorporates the neonate’s age and any past risk scores, and produces a new score that could be tracked over time. Using this approach on each patient from the Warner et al. and Olm et al. datasets, we observed divergence of risk scores between the NEC-affected and unaffected patients (Fig. 6A). Importantly, we performed risk score calculations for each patient within the two datasets using a MIL model that had never encountered that patient’s data. In this way, we were able to simulate what the risk score would have been, over time, for each patient in our two datasets if our system had been used during the course of their NICU admission.

In order to determine a maximally predictive risk score threshold, we evaluated a range of cutoff values, comparing their relative sensitivities and specificities for NEC. Results of this analysis are shown in Additional file 5: Fig. S4. We determined that a risk score of 0.35 had optimal performance when applied to our dataset, maximizing accuracy with an average lead-time (i.e. time before disease onset that the risk score rose above threshold) of 8.3 days. Figure 6B, C show cumulative correct predictions in each dataset as a function of time, demonstrating statistically significant differences in prediction rates between affected and unaffected patients. Figure 6D shows the relative proportion of true and false positives and negatives—as well as lead-times for risk scores >0.35—for all patients in this study. Figure 6E shows test characteristics for our system. Across the two studies, using a risk cutoff of 0.35, we found an overall sensitivity of 86% and a specificity of 90%.

### Interpretation of the growing bag model reveals bacterial taxa that drive accurate NEC predictions

We sought to interpret the major drivers of the prediction inflections revealed through the growing bag analysis, reasoning that interpretability data from our model would add to the validity of our approach if it highlighted bacterial taxa that past work has shown are correlative with NEC. We also speculated that interpretability data might indicate previously unstudied bacterial taxa that may play a role in NEC pathogenesis, and therefore be useful for generating new hypotheses about how NEC develops.

To interpret NEC predictions from our system, we took advantage of the attention weight module within the MIL architecture. We identified the highest attention weight instance from each positive bag in our two datasets. We extracted these instances and removed all clinical metadata, such that only microbiome data was left in the feature set. Next we performed random forest analysis on these high-attention instances in order to determine microbial covariants with attention weights (Fig. 3D). We analyzed each level of the bacterial taxonomic tree independently and performed five independent rounds of training and testing.

Using only the highest attention weight instances, random forest analysis was able to discriminate NEC-affected from non-affected patients, yielding ROC curve areas of 0.86 and 0.79 for test sets from the Warner and Olm datasets, respectively (Additional file 6: Fig. S5).

**Fig. 7:**
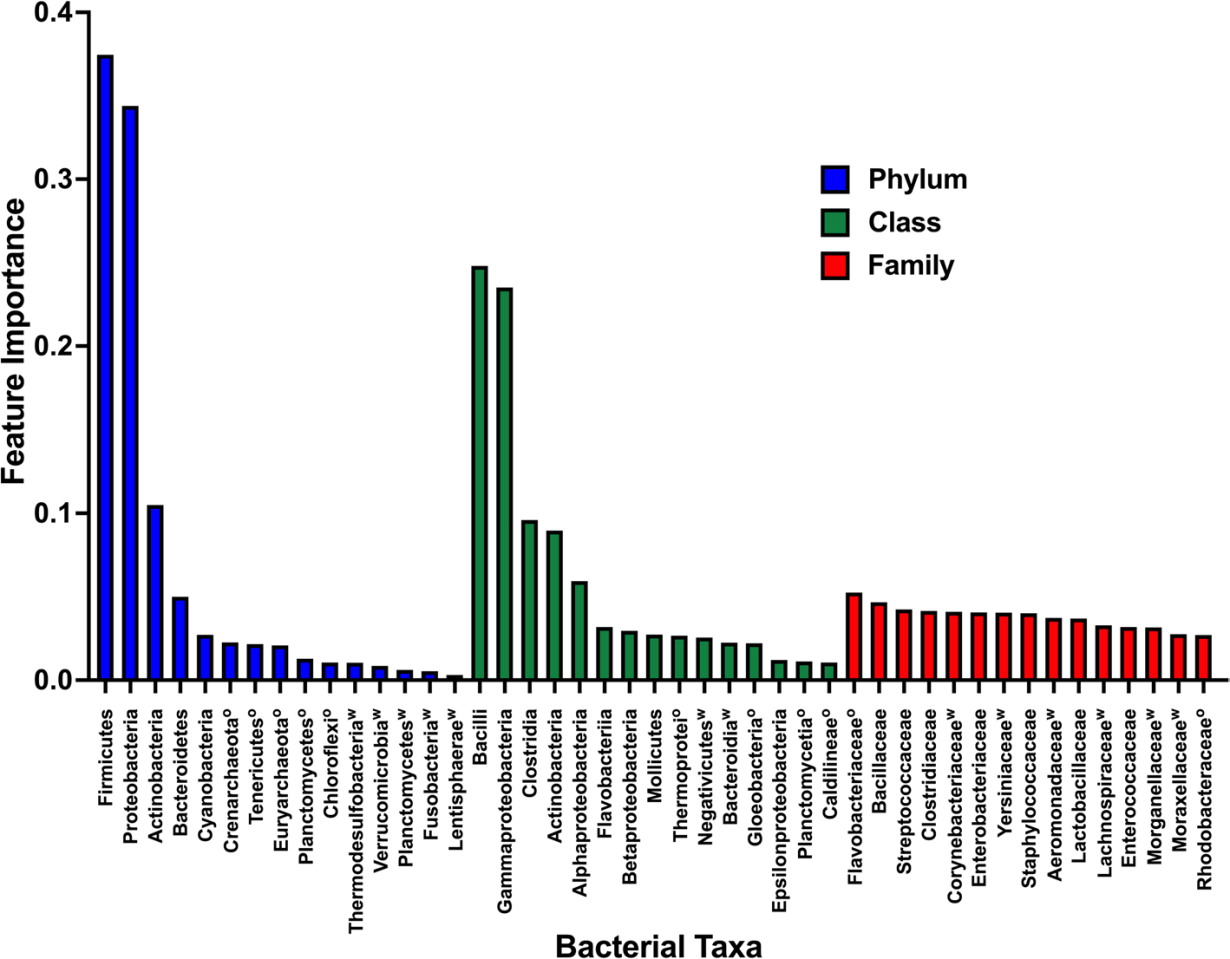
Feature importance based on analysis of high-attention weight instances. For each patient, the highest attention weight instance was identified and used to assess feature importance using random forest analysis. The 15 features with the highest scores are shown for the taxonomic levels of phylum, class, and family. Features that appeared in analyses from both studies are displayed as mean importance, while features that appeared in only one study are indicated with a superscript *w* for Warner et al. or *o* for Olm et al. For complete feature importance lists from both studies see Additional file 7: Data S2

Ranked importance values for the highest-influence features, shown in Fig. 7, demonstrate congruence between our results (shown for bacterial phylum, class, and family levels) and those reported by Warner and Olm et al. As in those studies, we found that phyla Firmicutes and Proteobacteria, class Gammaproteobacteria, and family Enterobacteriaceae were at or near the top of the importance-ranked feature lists for each taxonomic level we evaluated. Complete feature importance rankings from both studies are provided in Additional file 7: Data S2.

Interestingly, in our analysis some features emerged as important in one study dataset but not the other. For example, Flavobacteriaceae appeared on the list of important families from the Olm dataset, but not Warner (Fig. 7). This may reflect technical differences between how the two datasets were generated (for example, the divergent methods of next-generation sequencing), such that sensitivity for these taxa was higher in one study than the other. Alternatively, these differences may represent intrinsic aspects of the separate study populations. NEC pathogenesis may be geographically—as well as individually—heterogeneous, and interpretation differences between the two datasets may reflect this heterogeneity.

In addition to identifying previously suspected bacterial contributors to NEC pathogenesis, our analyses also highlight additional potentially important taxa. Classes Clostridia and Actinobacteria, for instance, were third- and fourth-highest importance (after Bacilli and Gammaproteobacteria), raising the possibility that these less-explored bacterial taxa may be worth further investigation in future studies.

## Discussion

We have developed and validated a ML system for converting serial measurements of bacterial taxa into a NEC risk score that could be tracked over time, potentially permitting earlier interventions to prevent the worst complications of the disease.

Multiple prior studies have investigated the developing neonatal intestinal microbiome, both in preterm and term populations, and several groups have focused on the preterm intestinal microbiota among patients who developed NEC [27–31, 58]. Most studies of the neonatal intestinal microbiota have relied on next-generation sequencing of bacterial DNA isolated or amplified from infant stool samples, while a subset have characterized bacteria from DNA isolated from surgical tissue samples [59–61].

These investigations have revealed a patterned progression in the establishment and subsequent evolution of the neonatal intestinal microbiota, while also demonstrating that there is significant individual variability. An early study of the neonatal microbiota by Dominguez-Bello et al. used data from multiple neonatal and maternal anatomic swab sites to demonstrate that the bacterial population characteristics of the neonatal microbiome in the immediate newborn period was significantly dependent on the mode of delivery, with patients born vaginally developing early microbiota resembling the maternal vaginal microbiome, while those delivered by Cesarean section developing early microbiota more closely resembling the population of maternal skin colonizers [20]. Over time, however, the microbiome profiles of different anatomic sites on the infant diverge and become increasingly site-dependent [25, 62, 63]. Subsequent work by Chu et al. demonstrated this and also showed that there is a microbiota associated with meconium, suggesting that the fetal intestine may not be completely sterile [64].

As the infant develops, stereotyped changes occur in the intestinal microbiota. Broadly speaking, early colonization by predominately aerobic bacteria is gradually replaced by anaerobic colonizers, while overall diversity increases with time [19, 21]. These patterned population shifts involve significant variation from individual to individual, however. The intestinal microbiota of two infants might follow dramatically different pathways to evolve from an early, aerobe-heavy state to a more diverse and anaerobic mature state. Common early life events such as viral infections, dietary changes, and administration of antibiotics can cause sudden shifts in an infant’s microbiome profile, and may temporarily perturb typical microbiota maturation [19]. Given time, however, most infants will reconstitute a normal microbiome and resume a generally predictable process of microbiome development.

Part of the challenge of trying to predict the occurrence of NEC in advance from microbiota data is that there does not seem to be a single common pathway to the disease. While the intestinal microbiota of infants affected by NEC show trends toward decreased overall diversity relative to unaffected controls [28], bacterial populations fluctuate over time and diversity can drop without signaling impending disease [19]. No single bacterial taxon nor group of taxa represents a necessary or sufficient cause of NEC. Instead, it is likely that different bacterial consortia can activate the inflammatory cascades necessary to trigger NEC under the proper conditions [27, 65].

NEC likely has multiple interacting causes, among which intestinal dysbiosis is only one contributor. Other factors that contribute to the pathophysiology of NEC include intestinal immaturity, bowel stasis, imbalance in microvascular tone, and a highly immunoreactive intestinal mucosa in preterm infants [66].

NEC therefore develops within a context of numerous, simultaneous, variable, and often non-linear microbiota changes. Different patterns may be associated with increased disease risk, and certain features of the data space—such as the sparsity and skewed distribution of microbial populations—make identifying a reliable signal challenging using traditional statistics. It is even possible that, at a mechanistic level, NEC is not a single disease entity but actually represents a final common pathway originating from multiple, distinct forms of underlying bowel pathology. This degree of complexity and uncertainty can stymie discovery approaches premised on a tight cause-and-effect relationship between a specific patient risk factor or clinical finding and disease occurrence.

This type of challenge can often be resolved through semi-supervised ML. MIL, in particular, is a semi-supervised ML approach well-suited to disease prediction from serial stool microbiota analyses. In this case, the overall task of assigning a probability of NEC must be completed using individual, complex instances (the sample analyses) that are not inherently NEC-positive or NEC-negative. This describes a MIL problem, and our work has shown that MIL can outperform alternative statistical analyses, establishing a reliable indicator of an infant’s NEC risk.

An advantage of our approach is that it can flexibly incorporate common events in a preterm infant’s NICU course that might present serious challenges to traditional statistics. Although infants in both the Warner and Olm studies experienced a range of postnatal cardiopulmonary depression (as indicated by their Apgar scores) and days exposed to antibiotics, received various forms of breathing support, and were fed breast milk and formula in differing proportions, none of these details were necessary as input into our MIL-based system, despite the fact that they all have the potential to disturb the progression of intestinal bacterial colonization. Instead, through ML training on adequate sample populations, the downstream effects of these clinical perturbations became incorporated into our model and did not affect its ability to determine an infant’s likelihood of developing NEC.

Our work also contributes a new application of MIL: the growing bag analysis, which we developed to incorporate passing time into our predictions, permitting establishment of a dynamic confidence score—essentially a time-dependent prediction of whether or not the patient, represented as a bag of instances enlarging over time, will develop NEC.

The growing bag approach revealed unexpected, sudden probability inflections that distinguished infants who would go on to develop NEC from those who would remain unaffected. The MIL system apparently identified a sudden and distinct change in NEC probability during the interval where the inflection appeared. These inflection points, in turn, allowed us to identify moments of changing risk, which could be directly translated into an easily comprehensible, trackable risk score. We speculate that this approach might form the basis of a practical bedside or clinical laboratory test for real-time determination of NEC risk among premature infants. Future work will also be devoted toward ascertaining what underlying microbiota changes drive the sudden confidence inflections among unaffected infants, as these factors may point the way toward better understanding of protective microbiome characteristics.

Our strategy also incorporated basic clinical and demographic metadata (Table 1). Certain traits, such as male gender and growth restriction, have been shown to be associated with higher NEC risk [67–69]. Furthermore, basic details such as these are readily available to any neonatology team caring for a preterm infant. Incorporating them into a bedside test would not require significant additional effort. As shown in Fig. 4, however, the clinical details shared between the Warner and Olm studies add only incrementally to our system’s predictive accuracy, which rests mostly on analysis of the microbiota. Whether there is core set of demographic and clinical metadata that significantly boosts the accuracy of MIL NEC risk prediction remains a topic for additional future study. We also speculate that high-resolution, continuously-recorded physiologic data—such as is used for prediction of sepsis from heart rate variability [70]—might enhance the predictive accuracy of our MIL system. Neither of the datasets we used for this study included this kind of physiologic tracing data. Similarly, it would potentially be informative to apply the machine learning strategies outlined here to metabolomic and proteomic datasets, either alone or in concert with microbiota data. Doing so might clarify underlying disease mechanisms and provide an avenue to novel biomarker discovery.

The attention-based pooling module within our system’s architecture adds an important dimension to this work. The attention assignments allow identification of instances within each bag that contribute most to the final bag label. Early proof-of-concept experiments with attention-based pooling demonstrated that it could be used to correctly identify target handwritten numerals. A similar algorithm has been used for cancer diagnosis from histopathology slides, a task that shares characteristics with microbiota analysis insofar as the key is often a subtle, variable change against a complex, noisy background. In this task, the highest attention went to regions of cellular dysplasia on the slides, indicating appropriate discrimination between high- and low-information components of the dataset [53]. Using attention weights in NEC prediction allowed us to proportionally amplify or suppress each stool sample’s influence on an infant’s risk score based on the system’s determination of that sample’s importance.

An interpretability analysis revealed that many of the taxa that had the greatest impact on our model’s predictions have been previously linked to NEC through metagenomic studies. Pammi et al. recompiled metagenomic data from a survey of methodologically sound investigations of the NEC-related intestinal microbiota. This group’s meta-analysis of these studies found significant NEC-related differences in the abundances of the Proteobacteria and Firmicutes phyla identified in our interpretability analysis [71]. This lends external validation to our approach and also supports the conclusions of other authors that these bacterial taxa contribute to development of NEC. It also suggests that by analyzing our ML model, we may be able to identify previously unrecognized bacterial taxa that contribute to NEC pathogenesis. This discovery approach might be hypothesis-generating for future basic and translational science studies to understand causes of NEC.

We believe the main contribution of this work to the fields of neonatology and machine learning is a MIL-based system that generates a longitudinal NEC risk score from a limited set of bacterial taxa and basic clinical metadata. The risk score algorithm treats each infant as a bag of instances, growing over time, and incorporates the system’s current confidence that the infant will develop NEC based on shifting bacterial taxa abundances and clinical information to date. It also utilizes information about past states of the growing bag; the attention weight given to each sample; the time that has passed since the last prediction was made; and how much time has passed since the infant’s birth in order to generate a longitudinal NEC risk score. Other diseases whose onset or severity correlates with complex microbiota population changes might be amenable to the same basic approach. Possible examples include inflammatory bowel disease, irritable bowel syndrome, colon cancer, and psychiatric illnesses. We anticipate evaluating our system’s ability to predict these conditions in future studies. We are also considering possible technical approaches to implementing repeated microbiota characterization of neonatal stool samples. We hope to conduct prospective validation studies in the near future, sequencing a novel collection of patient samples to generate new microbiota data for ML analysis. New technologies have been developed for rapid DNA purification and sequencing that do not require large, complex instrumentation [72, 73]. These technologies, combined with our ML approach, may present an avenue toward practical, local, ongoing NEC risk assessment for premature infants.

If NEC risk could be assessed and tracked, what would be the appropriate response to an asymptomatic infant whose risk was found to be rising? Since no reliable form of NEC risk prediction has been widely used, there is not a definite answer to this question; establishing an appropriate clinical response to a new diagnostic test takes time and experience, often in the form of additional clinical trials. A few plausible approaches to a rising NEC risk score include suspending enteral feeds (while giving supplementary IV nutrition), administering antibiotics, providing a prebiotic or a probiotic, or some combination of the above. None of these interventions are especially invasive, but would require further study and optimization in order to determine which (if any) has a beneficial approach on NEC risk. Perhaps one of the most immediate benefits of having a trackable NEC risk score would be an avenue toward additional clinical research on any of these interventions where the risk score could serve both as a trigger for randomization and as an outcome measure other than NEC or death. This would facilitate patient enrollment and potentially improve the statistical power of future studies.

## Conclusion

We have developed a novel, interpretable, MIL-based system for predicting NEC risk in preterm infant populations using data that can be collected non-invasively from otherwise discarded stool samples. Future work will involve prospective validation of this approach in hopes of developing a reliable method for predicting NEC before severe injury or death becomes unavoidable.

## Methods

### Data sources

After obtaining authorized access to clinical and demographic data through the National Center for Biotechnology Information (NCBI) Database of Genotypes and Phenotypes (dbGaP), we downloaded the raw sequence reads for 2,895 clinical samples from the Warner et al. study. For the Olm et al. dataset, we used the supplementary data from reference [27] to identify and download 700 sequence files from the NCBI Sequence Read Archive that could be matched to patients from the study whose NEC outcome was known.

We also included ten clinical metadata features collected and reported in both of the two historical studies (see Table 1). Clinical metadata present in one but not the other studies were not utilized.

### Kraken2 characterization of preterm infant stool microbiota

We used Kraken2 [41] to directly align the sequence reads to a database of microbiome DNA sequences (the miniKraken2 database), after which viral, fungal, and archaeal alignments were removed, leaving only bacterial matches.

### Validation of bacterial classification by confirming key findings from the initial studies

We validated key findings from the initial studies by comparing the kernel density plots of specific taxa of interest between the affected and unaffected patients. We conducted Kolmogorov–Smirnov tests on the kernel density plots to determine whether taxa distribution differences were statistically significant. Let $(x_1, x_2, ..., x_n)$ be samples of taxa abundance drawn from some unknown probability density function *f* at any given point *x*. We estimate *f* with $\hat{f}$ using kernel density estimator.

1 $$\begin{aligned} \hat{f}_h (x) = \frac{1}{nh} \sum _{i=1}^n K\big ( \frac{x - x_i}{h}\big ) \end{aligned}$$

where the bandwidth *h* is a smoothing parameter and *K* is the kernel which is a non-negative function. The kernel density estimator aims to smooth the population distribution curve given a finite set of samples. A histogram counts the number of data points at arbitrary regions; the kernel density estimator is the sum of the kernel function on every data point. For our analysis, we plotted kernel density estimator plots using the Gaussian kernel.

2 $$\begin{aligned} K\left( \frac{x - x_i}{h}\right) = \frac{1}{\sqrt{2 \pi }} e^{-\frac{1}{2} \left( \frac{x-x_i}{h}\right) ^2} \end{aligned}$$

### Hierarchical feature selection and center log-ratio transformation

In order to reduce the dimensionality, sparseness, and to correct for the compositional nature of our microbiota data, we performed several pre-processing steps. First, we represented the list of bacterial classifications reported in each Kraken2 output file as a taxonomic tree where related nodes (children) are linked with a common node (parent). This tree follows the traditional kingdom, phylum, class, order, family, group, species, strain taxonomic structure.

We performed a centered log-ratio transformation on each level of the taxonomic tree. The presence of zeros is problematic in computing the centered log-ratio transformation. Therefore, zeros were replaced with a uniform value: 0.66, which was 2/3 of the smallest possible read count of 1. Each taxon within a sample is transformed by taking the log-ratio counts for that taxon within a sample divided by the geometric mean of the counts of all taxa. Given an observation vector of D features in a sample, ${\mathbf {x}}=[x_1, x_2,...,x_D]$, the formulation for center log-ratio transformation ${\mathbf {x}}_{clr}$ is as follows:

3 $$\begin{aligned} {\mathbf {x}}_{clr}= & {} [log(x_1/G({\mathbf {x}})),log(x_2/G({\mathbf {x}})),...,log(x_D/G({\mathbf {x}})), ] \end{aligned}$$

4 $$\begin{aligned} G({\mathbf {x}})= & {} \root D \of {x_1 \cdot x_2 \cdot \cdots \cdot x_D} \end{aligned}$$

Finally, using a supervised feature selection approach similar to the hierarchical feature engineering (HFE) method [50], we reduced the dimensionality of our dataset by removing any node that was redundant with its parent node, using a Pearson correlation threshold of 0.7. This threshold allows for maintaining around $65\%$ of the original nodes for both datasets. We found 0.7 as an optimal threshold, since selecting a higher threshold would remove only a limited amount of nodes, while selecting a lower threshold would remove many child nodes that might contain information that is not captured by the parent node. Information gains were also calculated for each node of the taxonomy tree using the NEC target label. Any nodes with an information gain of zero was discarded.

### Development and benchmarking of the attention-based MIL system

In our MIL system for predicting NEC, we have a set of labeled examples:

$$(X_1, y_1), (X_2, y_2), \ldots , (X_n, y_n)$$

where each bag $X_i$ is a set of instances of the stool microbiota and clinical metadata features of the form $X_i = \{x_{i1}, x_{i2}, ..., x_{ik} \}$. Note that *k* corresponding to the number of samples can vary from one patient (bag) to another. The overall label $y_i \in \{ 0, 1 \}$ denotes the bag class label—that is, whether the baby developed NEC or not.

There is no access to the instance labels themselves. Therefore, the whole bag is labeled with $y_i = 1$, affected by NEC, if it includes at least one positive instance. The instances are considered weakly labeled because only a subset of them are the drivers of the prediction outcome. The bag label $y_i$ is given by:

5 $$\begin{aligned} y_i = {\left\{ \begin{array}{ll} 1,&{} \text {iff } \sum _{k} y_{ik}> 0 \\ 0, &{} \text {otherwise} \end{array}\right. } \end{aligned}$$

where $y_{ik}$ denotes the latent label of the *k*th instance of bag *i*. These $y_{ik}$ labels are not available during training while the bag label $y_i$ is observed.

The attention-based pooling function is constructed as follows. Obtaining the embedding $H = \{ h_1,...,h_k \}$ through a neural network $f_\psi (*)$, where $h_k = f_\psi (x_k)$, the embedding is aggregated by the weighted mean operator [53]:

6 $$\begin{aligned} {\mathbf {z}} = \sum _{k=1}^{k}a_{k}{\mathbf {h}}_{k} \end{aligned}$$

where

7 $$\begin{aligned} a_k = \frac{\exp \{{\mathbf {w}}^{\top }\tanh ({\mathbf {V}} {\mathbf {h}}_k^{\top })\} }{\sum _{j=1}^{k}\exp \{{\mathbf {w}}^{\top }\tanh ({\mathbf {V}} {\mathbf {h}}_j^{\top })\}} \end{aligned}$$

where ${\mathbf {w}} \in {\mathbb {R}}^{L \times 1}$ and ${\mathbf {V}} \in {\mathbb {R}}^{L \times M}$ are parameters.

Our system also includes a gated mechanism that has been shown to enhance MIL performance [53, 74].

8 $$\begin{aligned} a_k = \frac{\exp \{{\mathbf {w}}^\top (\tanh ({\mathbf {V}} {\mathbf {h}}_{k}^{\top }) \odot sigm({\mathbf {U}}{\mathbf {h}}_k^\top ))\}}{\sum _{j=1}^{k}\exp \{{\mathbf {w}}^\top (\tanh ({\mathbf {V}} {\mathbf {h}}_{k}^{\top }) \odot sigm({\mathbf {U}}{\mathbf {h}}_ k^\top ))\}} \end{aligned}$$

where ${\mathbf {U}} \in {\mathbb {R}}^{L \times M}$ are parameters and $\odot$ is an element-wise multiplication. The $sigm(\cdot )$ is a sigmoid function that serves to introduce non-linearity to the attention-based MIL pooling, improving efficiency in learning complex relationships that may be significant in determining the bag label. An overview of the computational architecture of our system is shown in Additional file 3: Fig. S2 inspired from [52].

### Growing bag analysis

Each patient is represented by a set of instances $X_{i} = \{x_{i1}, x_{i2}, ..., x_{ik}\}$, where *k* is the number of instances for that specific patient. A subset of instances $X_{i}^t \subseteq X_{i}$ where $X_{i}^t = \{x_{i1}, x_{i2}, ..., x_{it}\}$ can be used to represent the patient’s status at a fixed period in a patient’s life. We have $x_{i1}$ represent the first sample that was collected for a specific patient and $x_{it}$ as the *t*th sample. We define $D_{i} = \{d_{i1}, d_{i2}, ..., d_{ik}\}$ as the set of day-of-life values for a specific patient *i* and $D_{i}^t \subseteq D_{i}$, where $D_i^t = \{d_{i1}, d_{i2}, ..., d_{it}\}$. Using $X_{i}^t$ and $D_{i}^t$ as input into the MIL model, we can arrive at a scalar prediction confidence score $c_{it}$ and the attention weights $A_i^t = \{a_{i1},a_{i2}, ..., a_{it} \}$. Therefore, the set of prediction confidence $C_i^t = \{ c_{i1}, c_{i2}, ..., c_{it})$ can be obtained by iteratively applying the MIL model from $t=1$ to $t=k$ by using inputs $\{ (D_i^1, X_i^1), (D_i^2, X_i^2), ..., (D_i^t, X_i^t) \}$. We utilize the collection of prediction confidences and the attention weights for computing the risk score and conducting the interpretabilty analysis.

### Translating the growing bag into a trackable NEC risk score

For a specific patient *i*, the risk score is represented by $R_{i} = \{r_{i1}, r_{i2}, ..., r_{ik}\}$. The risk score calculated at each specific timepoint is dependent on the prior risk scores. We have noticed that for most non-affected patients the confidence score is high early in the patient’s life and then drops as more instances are added (see Fig. 5), while for the affected patients the confidence score stays close to 1. Based on this observation, our risk score formulation is constructed such that when there is a sharp decrease in the confidence score $(> 0.75)$ all subsequent instances’ contributions to the risk score become negative. We measure the contribution value using:

9 $$\begin{aligned} (d_{it} - d_{i (t-1)} )(c_{it} - 0.5) * (a_{it} - a_{it}^{min}) \end{aligned}$$

Instances in our dataset are not collected at a fixed interval. Therefore, we adjust the contribution value with the difference in the day-of-life for instance *t* and $t-1$ ($d_{it} - d_{i (t-1)}$). Confidence scores from our predictive model range from [0, 1], where a score of 0.5 is an indication of a random prediction. Intuitively, we zero-centered the confidence score $(c_{it} - 0.5)$ to reflect the idea that a random prediction should have no contribution to the risk score while a confidence score greater or less than 0.5 should have a positive or negative impact on the risk score, respectively. Given that the attention-based model allows an understanding of the contribution of each instance to a specific prediction, we factor in the marginal impact of including a new instance by looking at the difference between the newly included instance attention weight ($a_t$) and the minimum attention weight ($a_{it}^{min}$) in the bag.

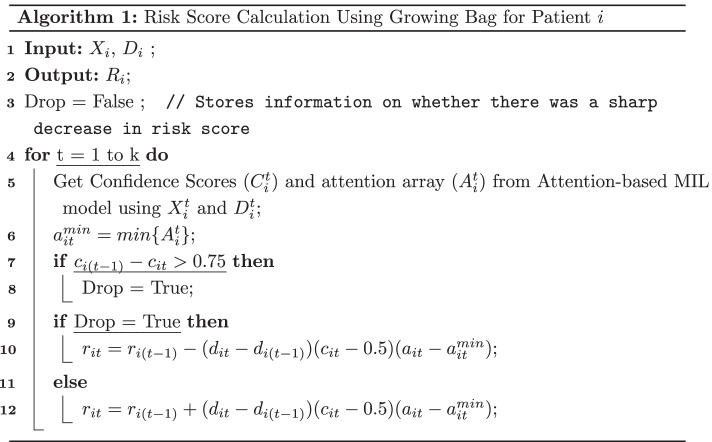



### Interpretability analysis using highest attention instances

In order to better understand the key features driving accurate predictions from the MIL attention model, we used the attention scores generated for each bag, which were used to isolate the most informative instances for analysis through a random forest algorithm. In the MIL model, bag labels were assigned using an aggregate of all instances, which were weighted by their attention scores as defined in (6). In this system, higher attention score instances contribute more to the final prediction. When the attention weight is zero the effect is essentially the same as removing the instance. Selecting the instance with the highest attention score to represent each patient simplifies the process of quantifying the feature importance of bacterial taxa in the microbiota, which was not allowed in the MIL model. With each patient represented as the highest attention weight instance, we used a traditional ML method to analyze feature importance.

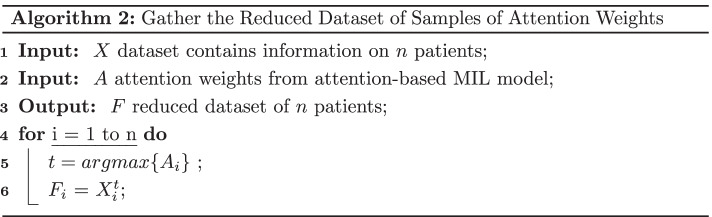



We trained a random forest model with the highest attention weight instances and extracted the importance for each of the features. We used the Scikit-Learn package to implement the random forest model and extract feature importances [57].

Random forest prediction results were gathered for the same train and test sets as in the original cross-validation of the MIL model (Additional file 6: Fig. S5).

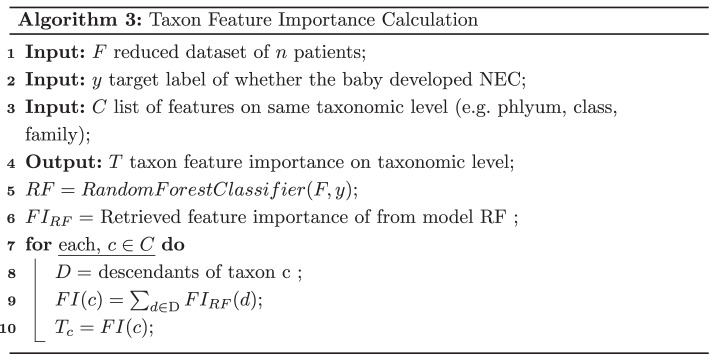



We summarized the feature importance within taxonomic levels of phylum, class, and family by summing the feature importances of descendent taxa with the same common ancestor.

The feature importance of a taxon at a specific taxonomic level (*FI*(*c*)) is calculated using Eq. (10).

10 $$\begin{aligned} FI(c) = \sum _{d \in {\text {D}}} FI_{RF}(d) \end{aligned}$$

*c* is a taxon on the taxonomic level of interest, *D* is the set of features generated after HFE selection and are descendants of *c* on the taxonomic tree.

The impurity-based feature importance ($FI_{RF}(d)$) is calculated for a random forest model by taking the average of all feature importance values from all trees in a random forest model (*T*).

11 $$\begin{aligned} FI_{RF}(d) = \frac{1}{|T|} \sum _{t \in T} FI_{DT}(d, t) \end{aligned}$$

Decision tree feature importance ($FI_{DT}(d, t)$) for a feature is the sum of information gain from all nodes that split on that feature, which we denote as $M_d$, divided by the total impurity reduction of all the nodes in tree *t*.

12 $$\begin{aligned} FI_{DT}(d, t) = \frac{\sum _{m \in M_d} IG(m)}{\sum _{f \in F} \sum _{m \in M_d} IG(m)} \end{aligned}$$

where *F* is the set of all features that was used to generate the random forest model.

Information gain is determined by impurity reduction achieved by splitting on features. Information gain on a node *m* is denoted as *IG*(*m*). There are several ways to calculate impurity (*I*(*m*)). The usual choice for classification is gini impurity, which is the method we used for our experiment.

13 $$\begin{aligned} IG(m) = I(m) - \left( \frac{N_m}{N_m^{right}} * I(m_\text {left}) + \frac{N_m}{N_m^{left}} * I({m_\text {right}}) \right) \end{aligned}$$

$N_m$ is the total number of samples in the current node, $N_m^{right}$ is the total number of samples is the right child node, and $N_m^{left}$ is the number of samples in the left. $m_{right}$ denotes the right child and $m_{left}$ denotes the left child.

## Supplementary Information

**Additional file 1: Figure S1**. Analyses of specific bacterial taxa abundances after Kraken2 classification and centered log-ratio transformation showed significant differences between NEC affected and non-affected patients that matched findings in the original Warner et al. and Olm et al. reports.

**Additional file 2: Data S1**. Complete lists of taxa included in models after pre-processing.

**Additional file 3: Figure S2**. A schematic of our MIL system for NEC risk prediction and interpretation. Diagram adapted from reference [52] (FCN, fully convolutional network; CLRT, centered log-ratio transformation; HFS, hierarchical feature selection).

**Additional file 4: Figure S3**. ROC and precision-recall curves generated from study data without centered log-ratio transformation. Input data for this figure did undergo hierarchical feature selection, without which the model failed to converge.

**Additional file 5: Figure S4**. Selection of the risk score cut-off of 0.35 was based on maximization of correct predictions among affected and non-affected patients.

**Additional file 6: Figure S5**. ROC and precision-recall curves generated from random forest analysis on only the MIL highest-attention instances. These curves represent five independent train-test replicates. The curve line illustrates the mean results from those five replicates. Error bars show 95% confidence intervals. Output from this analysis was used for the feature importance interpretability portion of the study.

**Additional file 7: Data S2**. Taxa importance ranks after interpratability analysis.

## Data Availability

Code and publicly available next-generation sequence accession numbers for all analyses are on GitHub at https://github.com/necdreamteam/NEC. Clinical metadata for the Warner et al. study may be obtained by permission from NCBI dbGAP (accession phs000247.v5.p3).

## Abbreviations

NEC: Necrotizing enterocolitis
MIL: Multiple instance learning
NICU: Neonatal intensive care unit
VLBW: Very low birth weight
ML: Machine learning
SVM: Support vector machine
ROC: Receiver-operator curve
AUC: Area under the curve
NCBI: National Center for Biotechnology Information
dbGaP: Database of Genotypes and Phenotypes

## References

[1] Alsaied A, Islam N, Thalib L (2020). Global incidence of necrotizing enterocolitis: a systematic review and Meta-analysis. BMC Pediatr.

[2] Neu J, Walker WA (2011). Necrotizing enterocolitis. N Engl J Med.

[3] Horbar JD, Carpenter JH, Badger GJ, Kenny MJ, Soll RF, Morrow KA, Buzas JS (2012). Mortality and neonatal morbidity among infants 501 to 1500 grams from 2000 to 2009. Pediatrics.

[4] Yee WH, Soraisham AS, Shah VS, Aziz K, Yoon W, Lee SK, Network CN (2012). Incidence and timing of presentation of necrotizing enterocolitis in preterm infants. Pediatrics.

[5] Fullerton BS, Hong CR, Velazco CS, Mercier CE, Morrow KA, Edwards EM, Ferrelli KR, Soll RF, Modi BP, Horbar JD, Jaksic T (2018). Severe neurodevelopmental disability and healthcare needs among survivors of medical and surgical necrotizing enterocolitis: a prospective cohort study. J Pediatr Surg.

[6] Wadhawan R, Oh W, Hintz SR, Blakely ML, Das A, Bell EF, Saha S, Laptook AR, Shankaran S, Stoll BJ (2014). Neurodevelopmental outcomes of extremely low birth weight infants with spontaneous intestinal perforation or surgical necrotizing enterocolitis. J Perinatol.

[7] Shah T, Meinzen-Derr J, Gratton T, Steichen J, Donovan E, Yolton K, Alexander B, Narendran V, Schibler K (2012). Hospital and neurodevelopmental outcomes of extremely low-birth-weight infants with necrotizing enterocolitis and spontaneous intestinal perforation. J Perinatol.

[8] Shah J, Singhal N, Silva Od, Rouvinez-Bouali N, Seshia M, Lee SK, Shah PS, Network CN (2015). Intestinal perforation in very preterm neonates: risk factors and outcomes. J Perinatol.

[9] Hull MA, Fisher JG, Gutierrez IM, Jones BA, Kang KH, Kenny M, Zurakowski D, Modi BP, Horbar JD, Jaksic T (2014). Mortality and management of surgical necrotizing enterocolitis in very low birth weight neonates: a prospective cohort study. J Am Coll Surg.

[10] Han SM, Knell J, Henry O, Riley H, Hong CR, Staffa SJ, Modi BP, Jaksic T (2020). Long-term outcomes of severe surgical necrotizing enterocolitis. J Pediatr Surg.

[11] Bazacliu C, Neu J (2019). Necrotizing enterocolitis: long term complications. Curr Pediatr Rev.

[12] Rich BS, Dolgin SE (2017). Necrotizing enterocolitis. Pediatr Rev.

[13] Dominguez KM, Moss RL (2012). Necrotizing enterocolitis. Clin Perinatol.

[14] Dermyshi E, Wang Y, Yan C, Hong W, Qiu G, Gong X, Zhang T (2017). The “golden age” of probiotics: a systematic review and meta-analysis of randomized and observational studies in preterm infants. Neonatology.

[15] Kona SK, Matlock DN (2018). Probiotics, prebiotics, and synbiotics for preterm neonates. NeoReviews.

[16] Sharif S, Meader N, Oddie SJ, Rojas-Reyes MX, McGuire W (2020). Probiotics to prevent necrotising enterocolitis in very preterm or very low birth weight infants. Cochrane Database Syst Rev.

[17] Valpacos M, Arni D, Keir A, Aspirot A, Wilde JC, Beasley S, De Luca D, Pfister RE, Karam O (2018). Diagnosis and management of necrotizing enterocolitis: an international survey of neonatologists and pediatric surgeons. Neonatology.

[18] Torrazza RM, Torrazza RM, Neu J (2011). The developing intestinal microbiome and its relationship to health and disease in the neonate. J Perinatol.

[19] Koenig JE, Spor A, Scalfone N, Fricker AD, Stombaugh J, Knight R, Angenent LT, Ley RE (2011). Succession of microbial consortia in the developing infant gut microbiome. Proc Natl Acad Sci U S Am.

[20] Dominguez-Bello MG, Costello EK, Contreras M, Magris M, Hidalgo G, Fierer N, Knight R (2010). Delivery mode shapes the acquisition and structure of the initial microbiota across multiple body habitats in newborns. Proc Natl Acad Sci.

[21] Palmer C, Bik EM, DiGiulio DB, Relman DA, Brown PO (2007). Development of the human infant intestinal microbiota. PLoS Biol.

[22] Brown CT, Xiong W, Olm MR, Thomas BC, Baker R, Firek B, Morowitz MJ, Hettich RL, Banfield JF (2018). Hospitalized premature infants are colonized by related bacterial strains with distinct proteomic profiles. mBio.

[23] Mai V, Torrazza RM, Ukhanova M, Wang X, Sun Y, Li N, Shuster J, Sharma R, Hudak ML, Neu J (2013). Distortions in development of intestinal microbiota associated with late onset sepsis in preterm infants. PLoS ONE.

[24] Eck A, Rutten NBMM, Singendonk MMJ, Rijkers GT, Savelkoul PHM, Meijssen CB, Crijns CE, Oudshoorn JH, Budding AE, Vlieger AM (2020). Neonatal microbiota development and the effect of early life antibiotics are determined by two distinct settler types. PLoS ONE.

[25] Human Microbiome Project Consortium (2012). Structure, function and diversity of the healthy human microbiome. Nature.

[26] Dave M, Higgins PD, Middha S, Rioux KP (2012). The human gut microbiome: current knowledge, challenges, and future directions. Transl Res J Lab Clin Med.

[27] Olm MR, Bhattacharya N, Crits-Christoph A, Firek BA, Baker R, Song YS, Morowitz MJ, Banfield JF. Necrotizing enterocolitis is preceded by increased gut bacterial replication, klebsiella, and fimbriae-encoding bacteria that may stimulate tlr4 receptors. bioRxiv. 10.1101/558676 (2019). 10.1126/sciadv.aax5727 PMC6905865 31844663

[28] Dobbler PT, Procianoy RS, Mai V, Silveira RC, Corso AL, Rojas BS, Roesch LF (2017). Low microbial diversity and abnormal microbial succession is associated with necrotizing enterocolitis in preterm infants. Front Microbiol.

[29] Mai V, Young CM, Ukhanova M, Wang X, Sun Y, Casella G, Theriaque D, Li N, Sharma R, Hudak M, Neu J (2011). Fecal microbiota in premature infants prior to necrotizing enterocolitis. PLoS ONE.

[30] Gopalakrishna KP, Macadangdang BR, Rogers MB, Tometich JT, Firek BA, Baker R, Ji J, Burr AHP, Ma C, Good M, Morowitz MJ, Hand TW (2019). Maternal IgA protects against the development of necrotizing enterocolitis in preterm infants. Nat Med.

[31] Warner BB, Deych E, Zhou Y, Hall-Moore C, Weinstock GM, Sodergren E, Shaikh N, Hoffmann JA, Linneman LA, Hamvas A, Khanna G, Rouggly-Nickless LC, Ndao IM, Shands BA, Escobedo M, Sullivan JE, Radmacher PG, Shannon WD, Tarr PI (2016). Gut bacteria dysbiosis and necrotising enterocolitis in very low birthweight infants: a prospective case-control study. Lancet.

[32] Ji J, Ling XB, Zhao Y, Hu Z, Zheng X, Xu Z, Wen Q, Kastenberg ZJ, Li P, Abdullah F, Brandt ML, Ehrenkranz RA, Harris MC, Lee TC, Simpson BJ, Bowers C, Moss RL, Sylvester KG (2014). A data-driven algorithm integrating clinical and laboratory features for the diagnosis and prognosis of necrotizing enterocolitis. PLoS ONE.

[33] Lugli GA, Milani C, Mancabelli L, Turroni F, Sinderen Dv, Ventura M (2019). A microbiome reality check: limitations of in silico-based metagenomic approaches to study complex bacterial communities. Environ Microbiol Rep.

[34] Gloor GB, Wu JR, Pawlowsky-Glahn V, Egozcue JJ (2016). It’s all relative: analyzing microbiome data as compositions. Ann Epidemiol.

[35] Gloor GB, Macklaim JM, Pawlowsky-Glahn V, Egozcue JJ (2017). Microbiome datasets are compositional: and this is not optional. Front Microbiol.

[36] Martino C, Morton JT, Marotz CA, Thompson LR, Tripathi A, Knight R, Zengler KA (2019). Novel sparse compositional technique reveals microbial perturbations. mSystems.

[37] Hooven T, Lin YC, Salleb-Aouissi A. Multiple instance learning for predicting necrotizing enterocolitis in premature infants using microbiome data. In: Proceedings of the ACM conference on health, inference, and learning, CHIL ’20. New York: Association for Computing Machinery; 2020. pp. 99–109. 10.1145/3368555.3384466 10.1145/3368555.3384466 PMC8313028 34318306

[38] Foulds J, Frank E (2010). A review of multi-instance learning assumptions. Knowl Eng Rev.

[39] Ranjan R, Rani A, Metwally A, McGee HS, Perkins DL (2016). Analysis of the microbiome: advantages of whole genome shotgun versus 16S amplicon sequencing. Biochem Biophys Res Commun.

[40] Brumfield KD, Huq A, Colwell RR, Olds JL, Leddy MB (2020). Microbial resolution of whole genome shotgun and 16S amplicon metagenomic sequencing using publicly available NEON data. PLoS ONE.

[41] Wood DE, Lu J, Langmead B (2019). Improved metagenomic analysis with Kraken 2. Genome Biol.

[42] Kumar MS, Slud EV, Okrah K, Hicks SC, Hannenhalli S, Bravo HC (2018). Analysis and correction of compositional bias in sparse sequencing count data. BMC Genomics.

[43] Egozcue JJ, Pawlowsky-Glahn V (2019). Compositional data: the sample space and its structure. TEST.

[44] Aitchison J (1984). Reducing the dimensionality of compositional data sets. J Int Assoc Math Geol.

[45] Mandal S, Van Treuren W, White RA, Eggesbø M, Knight R, Peddada SD (2015). Analysis of composition of microbiomes: a novel method for studying microbial composition. Microbial Ecol Health Dis.

[46] Xia F, Chen J, Fung WK, Li H (2013). A logistic normal multinomial regression model for microbiome compositional data analysis. Biometrics.

[47] Egozcue JJ, Pawlowsky-Glahn V, Mateu-Figueras G, Barcelo-Vidal C (2003). Isometric logratio transformations for compositional data analysis. Math Geol.

[48] Aitchison J. The statistical analysis of compositional data. Chapman & Hall Ltd., GBR; 1986.

[49] Qu K, Gao F, Guo F, Zou Q (2019). Taxonomy dimension reduction for colorectal cancer prediction. Comput Biol Chem.

[50] Oudah M, Henschel A (2018). Taxonomy-aware feature engineering for microbiome classification. BMC Bioinform.

[51] Dietterich TG, Lathrop RH, Lozano-Perez T (1997). Solving the multiple instance problem with axis-parallel rectangles. Artif Intell.

[52] Carbonneau M, Cheplygina V, Granger E, Gagnon G. Multiple instance learning: a survey of problem characteristics and applications. CoRR. arxiv: abs/1612.03365 (2016).

[53] Ilse M, Tomczak JM, Welling M. Attention-based deep multiple instance learning. In: Proceedings of the 35th international conference on machine learning, ICML 2018, Stockholmsmässan, Stockholm, Sweden, July 10-15, 2018, pp. 2132–2141. http://proceedings.mlr.press/v80/ilse18a.html (2018).

[54] Andrews S, Tsochantaridis I, Hofmann T. Support vector machines for multiple-instance learning. In: Advances in neural information processing systems; 2003. pp. 577–584

[55] Zhang C, Platt JC, Viola PA Multiple instance boosting for object detection. In: Advances in neural information processing systems; 2006. pp. 1417–1424.

[56] Paszke A, Gross S, Massa F, Lerer A, Bradbury J, Chanan G, Killeen T, Lin Z, Gimelshein N, Antiga L, Desmaison A, Köpf A, Yang E, DeVito Z, Raison M, Tejani A, Chilamkurthy S, Steiner B, Fang L, Bai J, Chintala S. Pytorch: an imperative style, high-performance deep learning library. CoRR. arxiv: abs/1912.01703 (2019).

[57] Pedregosa F, Varoquaux G, Gramfort A, Michel V, Thirion B, Grisel O, Blondel M, Prettenhofer P, Weiss R, Dubourg V (2011). Scikit-learn: machine learning in python. J Mach Learn Res.

[58] Raveh-Sadka T, Thomas BC, Singh A, Firek B, Brooks B, Castelle CJ, Sharon I, Baker R, Good M, Morowitz MJ, Banfield JF (2015). Gut bacteria are rarely shared by co-hospitalized premature infants, regardless of necrotizing enterocolitis development. eLife.

[59] Smith B, Bodé S, Petersen BL, Jensen TK, Pipper C, Kloppenborg J, Boyé M, Krogfelt KA, Mølbak L (2011). Community analysis of bacteria colonizing intestinal tissue of neonates with necrotizing enterocolitis. BMC Microbiol.

[60] Brower-Sinning R, Zhong D, Good M, Firek B, Baker R, Sodhi CP, Hackam DJ, Morowitz MJ (2014). Mucosa-associated bacterial diversity in necrotizing enterocolitis. PLoS ONE.

[61] Romano-Keeler J, Shilts MH, Tovchigrechko A, Wang C, Brucker RM, Moore DJ, Fonnesbeck C, Meng S, Correa H, Lovvorn HN, Tang Y-W, Hooper L, Bordenstein SR, Das SR, Weitkamp J-H (2018). Distinct mucosal microbial communities in infants with surgical necrotizing enterocolitis correlate with age and antibiotic exposure. PLoS ONE.

[62] Capone KA, Dowd SE, Stamatas GN, Nikolovski J (2011). Diversity of the human skin microbiome early in life. J Invest Dermatol.

[63] Mesa MD, Loureiro B, Iglesia I, Fernandez Gonzalez S, Llurba Olivé E, Garcia Algar O, Solana MJ, Cabero Perez M, Sainz T, Martinez L (2020). The evolving microbiome from pregnancy to early infancy: A comprehensive review. Nutrients.

[64] Chu DM, Ma J, Prince AL, Antony KM, Seferovic MD, Aagaard KM (2017). Maturation of the infant microbiome community structure and function across multiple body sites and in relation to mode of delivery. Nat Med.

[65] Rusconi B, Good M, Warner BB (2017). The microbiome and biomarkers for necrotizing enterocolitis: are we any closer to prediction?. J Pediatr..

[66] Petrosyan M, Guner YS, Williams M, Grishin A, Ford HR (2009). Current concepts regarding the pathogenesis of necrotizing enterocolitis. Pediatr Surg Int.

[67] Drenckpohl D, Knaub L, Schneider C, McConnell C, Wang H, Macwan K (2010). Risk factors that may predispose premature infants to increased incidence of necrotizing enterocolitis. ICAN Infant Child Adolesc Nutr.

[68] Carter BM, Holditch-Davis D (2008). Risk Factors for Necrotizing Enterocolitis in Preterm Infants. Adv Neonatal Care.

[69] Ito M, Tamura M, Namba F, Japan t.N.R.N.o. (2017). Role of sex in morbidity and mortality of very premature neonates. Pediatr Int.

[70] Fairchild K (2012). Aschner: HeRO monitoring to reduce mortality in NICU patients. Res Rep Neonatol.

[71] Pammi M, Cope J, Tarr PI, Warner BB, Morrow AL, Mai V, Gregory KE, Kroll JS, McMurtry V, Ferris MJ, Engstrand L, Lilja HE, Hollister EB, Versalovic J, Neu J (2017). Intestinal dysbiosis in preterm infants preceding necrotizing enterocolitis: a systematic review and meta-analysis. Microbiome.

[72] Soni S, Toley BJ (2022). Paper-based nucleic acid sample preparation for point-of-care diagnostics. Sens Actuators B Chem.

[73] Kai S, Matsuo Y, Nakagawa S, Kryukov K, Matsukawa S, Tanaka H, Iwai T, Imanishi T, Hirota K (2019). Rapid bacterial identification by direct PCR amplification of 16S rRNA genes using the MinION nanopore sequencer. FEBS Open Bio.

[74] Dauphin YN, Fan A, Auli M, Grangier D. Language modeling with gated convolutional networks. CoRR. arxiv: abs/1612.08083 (2016).

