# Supplementary figures and images for: Interpretable prediction of necrotizing enterocolitis from machine learning analysis of premature infant stool microbiota
Source: BMC Bioinformatics. 2022 Mar 25;23:104. doi: 10.1186/s12859-022-04618-w (PMC8953333; doi:10.1186/s12859-022-04618-w)

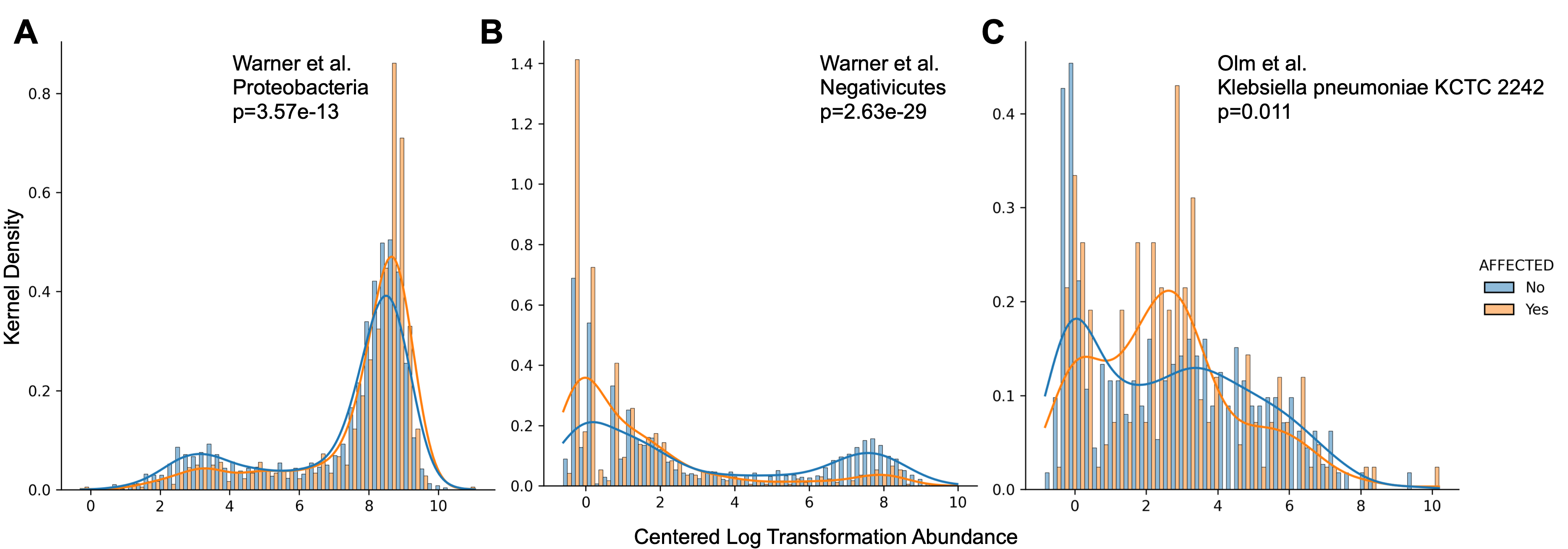

Supplement: Supplementary file 1 — Additional file 1: Figure S1. Analyses of specific bacterial taxa abundances after Kraken2 classification and centered log-ratio transformation showed significant differences between NEC affected and non-affected patients that matched findings in the original Warner et al. and Olm et al. reports. [file 12859_2022_4618_MOESM1_ESM.png]

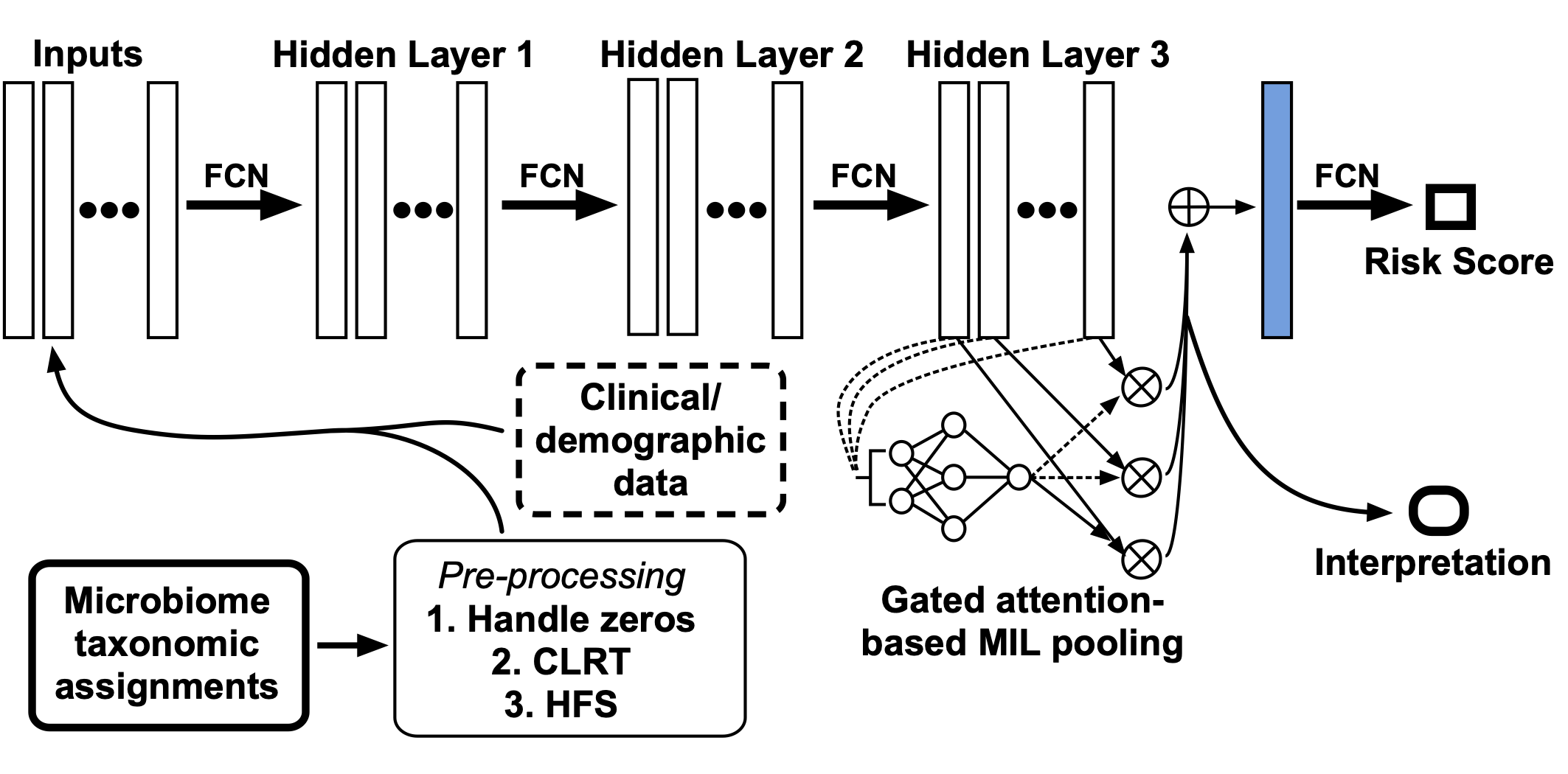

Supplement: Supplementary file 3 — Additional file 3: Figure S2. A schematic of our MIL system for NEC risk prediction and interpretation. Diagram adapted from reference [52] (FCN, fully convolutional network; CLRT, centered log-ratio transformation; HFS, hierarchical feature selection). [file 12859_2022_4618_MOESM3_ESM.png]

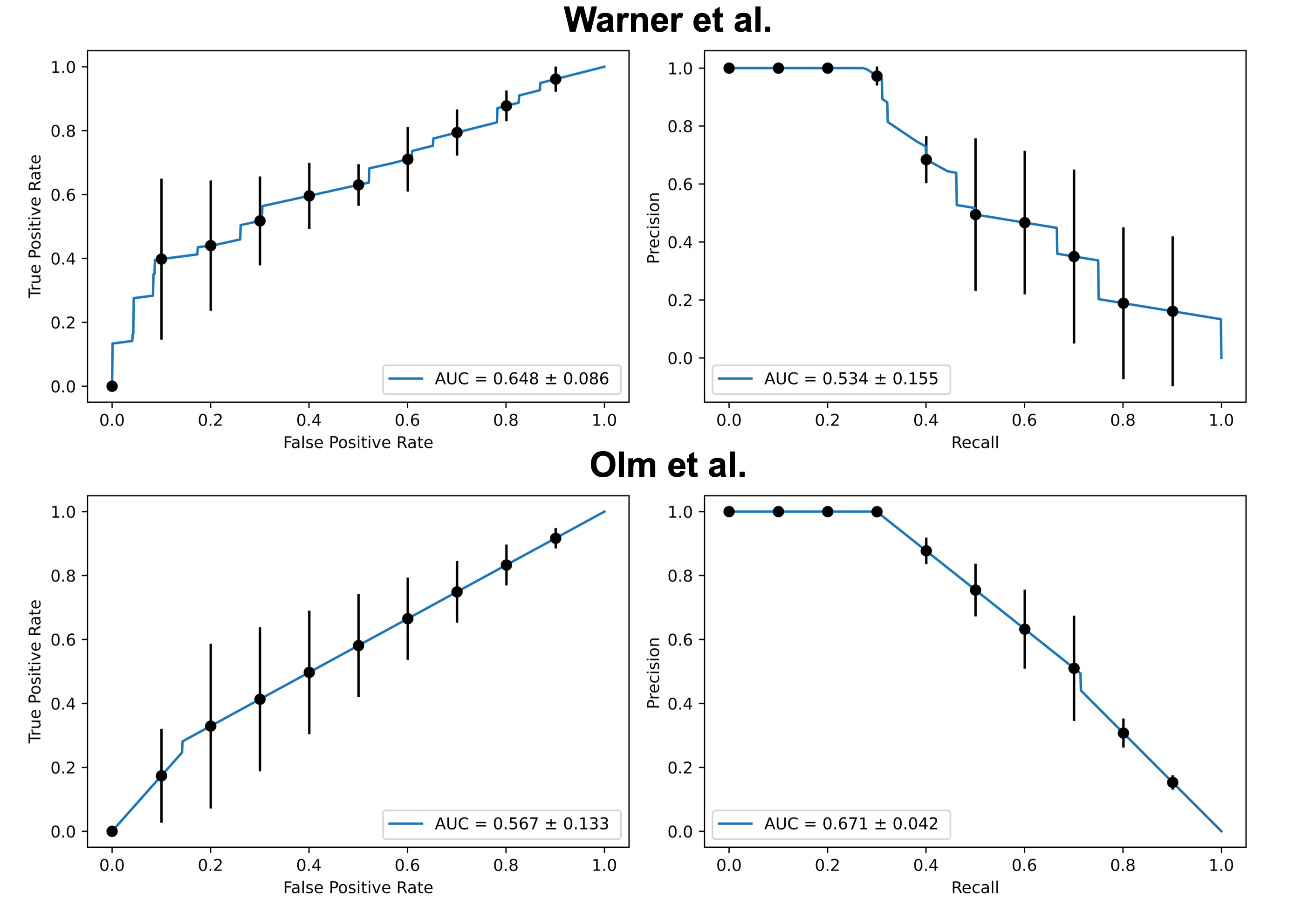

Supplement: Supplementary file 4 — Additional file 4: Figure S3. ROC and precision-recall curves generated from study data without centered log-ratio transformation. Input data for this figure did undergo hierarchical feature selection, without which the model failed to converge. [file 12859_2022_4618_MOESM4_ESM.png]

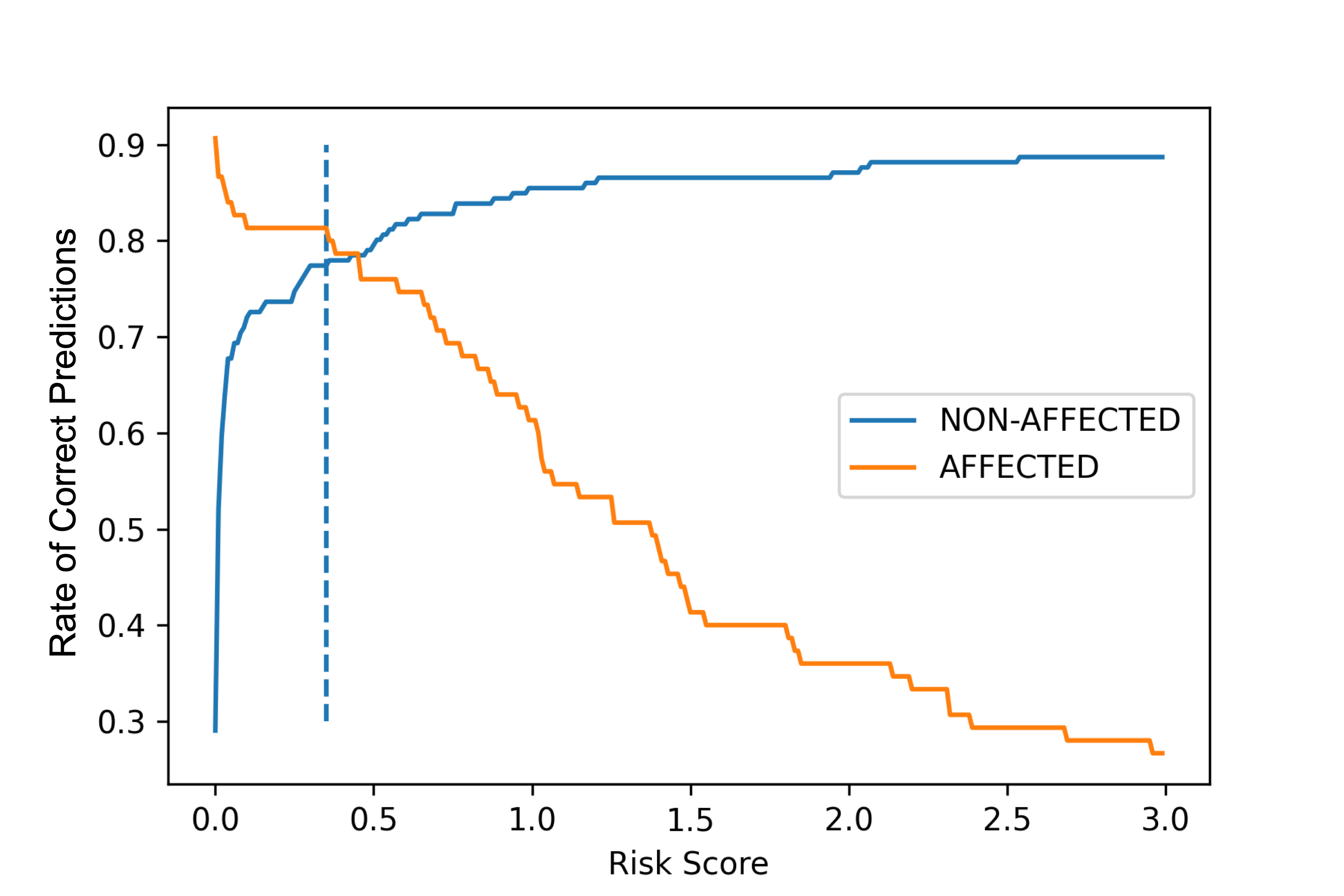

Supplement: Supplementary file 5 — Additional file 5: Figure S4. Selection of the risk score cut-off of 0.35 was based on maximization of correct predictions among affected and non-affected patients. [file 12859_2022_4618_MOESM5_ESM.png]

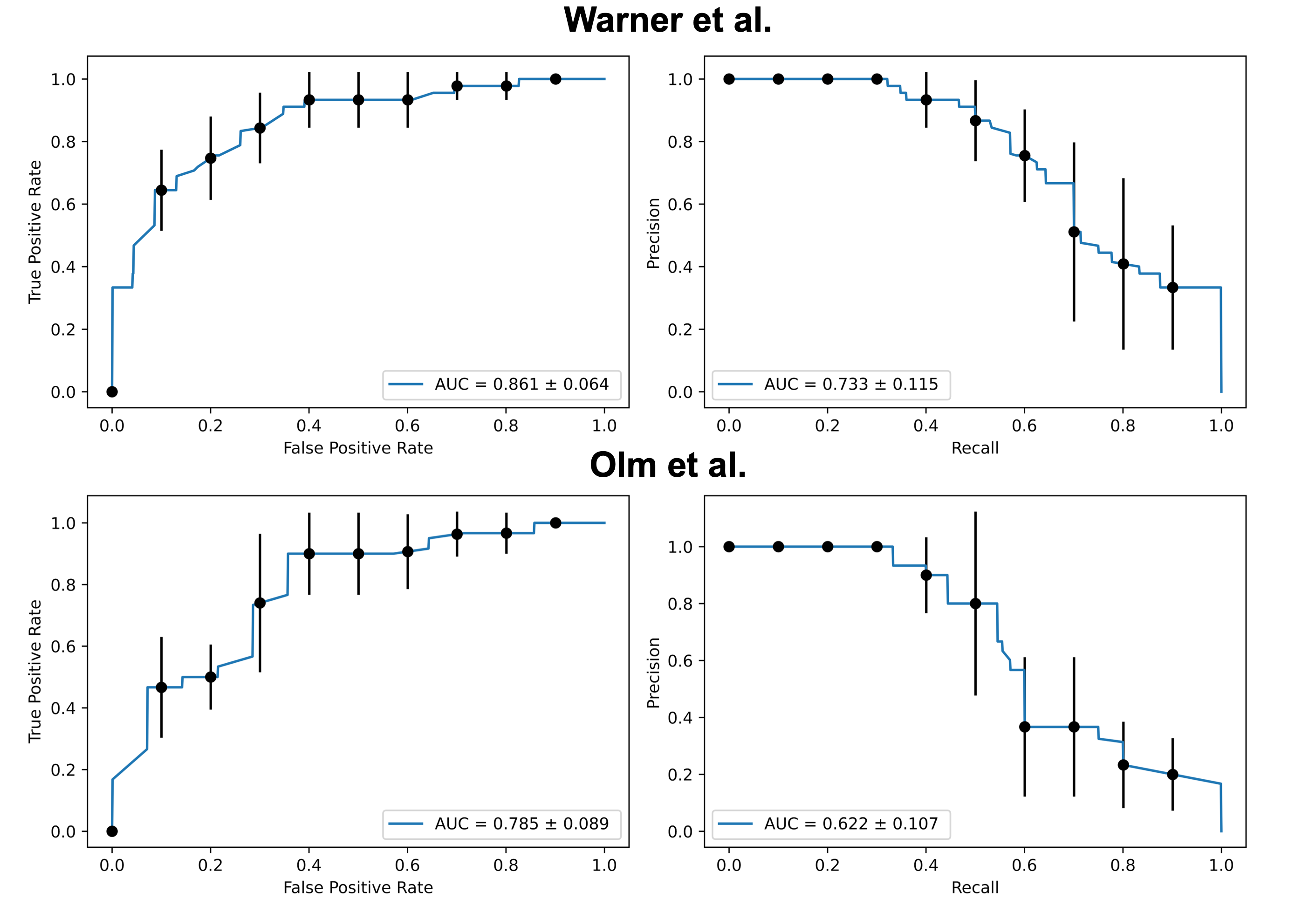

Supplement: Supplementary file 6 — Additional file 6: Figure S5. ROC and precision-recall curves generated from random forest analysis on only the MIL highest-attention instances. These curves represent five independent train-test replicates. The curve line illustrates the mean results from those five replicates. Error bars show 95% confidence intervals. Output from this analysis was used for the feature importance interpretability portion of the study. [file 12859_2022_4618_MOESM6_ESM.png]
